# Genomic traits, transcriptional responses, and metabolic activities determine functional divergence in mineral dissolution between *Priestia megaterium* PMA2-2 and *Priestia flexa* PMD1-1

**DOI:** 10.1128/aem.01201-26

**Published:** 2026-08-10

**Authors:** Wen Dong, Sheng-Zhi Zhang, Duo Yu, Qi Sheng, Lin-Yan He, Xia-Fang Sheng

**Affiliations:** 1 College of Life Sciences, Nanjing Agricultural University 70578, Nanjing, China; Colorado School of Mines, Golden, Colorado, USA

**Keywords:** *Priestia *strains, dissolution phenotype, transcription response, metabolic activity, functional divergence in mineral dissolution

## Abstract

*Priestia* strains are gram-positive bacteria and globally distributed in mineral-rich environments. However, the functional divergence in mineral dissolution and its molecular drivers among *Priestia* strains remains poorly understood. Here, we employ comparative phenotypic and multi-omics analyses to decipher the molecular mechanisms for divergent mineral dissolution phenotypes between the efficient mineral-dissolving *Priestia megaterium* PMA2-2 and the inefficient mineral-dissolving *Priestia flexa* PMD1-1. Compared to PMD1-1, PMA2-2 exhibited significantly enhanced biotite dissolution, medium acidification, polysaccharide production, and biofilm formation on the biotite surface. Genomic analysis revealed that PMA2-2 harbored more genes associated with organic acid metabolism, biofilm formation, and EPS biosynthesis than PMD1-1. Transcriptomic and metabolomic analyses linked PMA2-2’s enhanced biotite-dissolution phenotype to the coordinated upregulation of genes located on both its chromosome and plasmids, and enhanced production of exometabolites involved in organic acids, proton efflux, biofilm formation, polysaccharide production, amino acid metabolism, cell wall components, and flagellar assembly. Furthermore, genes involved in environmental sensing (including two-component systems and quorum sensing) and nutrient transport systems (ABC transporters) were also upregulated in PMA2-2. Genomic conservation analysis of PMA2-2 revealed that the genes responsible for efficient mineral dissolution are widespread and phylogenetically conserved across the *Priestia* genus. These core mineral dissolution-related genes were responsive to a range of silicate and oxide minerals (biotite, potassium-feldspar, olivine, and hematite). Our findings establish a genotype-to-phenotype map for mineral dissolution, providing new insights into the functional divergence in mineral dissolution between *Priestia* strains PMA2-2 and PMD1-1, as well as the molecular mechanisms that facilitate these processes.

**IMPORTANCE:**

To date, the molecular mechanisms underlying the functional divergence in mineral dissolution in gram-positive *Priestia* strains are largely unexplored. This study characterizes the mechanisms involved in functional divergence in biotite dissolution between two closely related strains of *Priestia*, one an efficient mineral-dissolving strain (PMA2-2) and the other an inefficient mineral-dissolving strain (PMD1-1). The coordinated upregulation of genes and exometabolites associated with organic acid and polysaccharide production, proton efflux, biofilm formation, cell wall components, as well as environmental sensing and nutrient transport systems may be responsible for PMA2-2’s enhanced biotite dissolution phenotype. The multiple genes associated with efficient mineral dissolution by PMA2-2 are widespread and conserved among *Priestia* strains. Furthermore, a range of silicate and oxide minerals induced the expression of these core genes related to mineral dissolution by PMA2-2. Our results provide new understandings concerning the molecular mechanisms responsible for driving functional divergence in mineral dissolution between these *Priestia* strains.

## INTRODUCTION

Bacteria-enhanced mineral dissolution is a core driver for geological evolution, linking the biosphere, lithosphere, and atmosphere, and regulating global geochemical processes and carbon cycling (1, 2). In terrestrial ecosystems, bacteria can adapt to nutrient-limited environments (1, 3) and play important roles in silicate mineral dissolution, soil formation, elemental biogeochemical cycling, and mitigation of climate change via carbon sequestration (4–6). However, a significant knowledge gap persists in understanding how specific genetic and regulatory architectures among closely related strains contribute to functional divergence in mineral weathering efficiency. This limits insights into the evolutionary process and ecological specialization of key weathering agents (3, 5). The geological functions of mineral dissolution by bacteria rely on multiple physiological and molecular mechanisms (5, 7). Three core strategies dominate: (i) acidolysis, involving the secretion of organic acids and proton efflux to decrease environmental pH (8–11), (ii) chelation, where organic acids and siderophores scavenge Fe(III) from mineral lattices (11, 12) and exopolysaccharides (EPS) form biofilms that enhance cell adsorption on mineral surfaces (13, 14); and (iii) adsorption, mediated by cell wall components that promote stable physical contact with minerals (10, 15, 16). Furthermore, two-component systems (TCS) sense environmental signals to trigger metabolic responses (15, 17–19), while ABC transporters can regulate nutrient uptake and substrate efflux (18–20). Moreover, master regulators, such as Spo0A encoded by *spoOA* (encoding the major early sporulation transcription factor) in Bacillales, centrally coordinate biofilm formation and metabolic shifts in bacteria (21, 22).

A combination of genomics, transcriptomics, and genetics in the mineral-dissolving bacterium *Pseudomonas azotoformans* F77 revealed the upregulated genes involved in gluconic acid metabolism, flagellar assembly, and pilus biosynthesis during the biotite dissolution process by this strain (15). Dual transcriptomics and proteomics demonstrated that the mineral-dissolving bacterium *Caballeronia mineralivorans* PML1(12) was capable of mobilizing iron through the production of a non-ribosomal peptide synthetase-independent siderophore and strongly acidifying its environment with glucose using a suite of glucose/methanol/choline oxidoreductases to weather biotite (18). Furthermore, a comparative transcriptomic analysis indicated that significantly upregulated differentially expressed genes in mineral-dissolving strain *Priestia aryabhattai* C4-10 were enriched in pathways related to glyoxylate and dicarboxylate metabolism, amino acid biosynthesis, the tricarboxylic acid cycle, and ABC transporters in the presence of biotite (23). However, the molecular mechanisms regulating silicate mineral dissolution in gram-positive *Priestia* strains remain poorly understood (7), hindering a comprehensive understanding of the interactions between silicate minerals and mineral-dissolving *Priestia* strains.

*Priestia*, formerly classified within the *Bacillus* genus, has been established as an independent genus (24). Mineral-dissolving *Priestia* strains are ubiquitous gram-positive bacteria at various geological interfaces, including rhizospheres, soil-rock boundaries, weathering crusts, and nutrient-poor rock surfaces, demonstrating a better adaptation to various environments (25, 26). Both *Bacillus* and *Priestia* strains can enhance mineral dissolution via the production of organic acids, EPS, siderophores, and biofilms (23, 25). However, studies on *Priestia* strains have primarily focused on the verification of mineral dissolution by single-strain approaches, lacking systematic comparisons of mineral dissolution phenotypes and the underlying molecular mechanisms between closely related *Priestia* strains with different levels of mineral dissolution capacity. More critically, it remains unknown whether the genetic basis for efficient mineral dissolution is conserved within *Priestia* strains or if strain-specific mineral dissolution advantages are acquired through mechanisms like plasmid transfer. In this study, we hypothesized that the efficient mineral-dissolving *P. megaterium* PMA2-2 possesses and coordinately expresses a core set of genes involved in organic acid and EPS production, biofilm formation, cell wall components, and environmental sensing, which are either absent or under-expressed in the inefficient mineral-dissolving *P. flexa* PMD1-1. We further proposed that this core set of genes is phylogenetically conserved and inducible by a range of silicate and oxide minerals, representing a generalized adaptation strategy within the *Priestia* genus.

This study aims to determine the difference in mineral dissolution capacity between two closely related *Priestia* strains PMA2-2 and PMD1-1 and whether the significant divergence in mineral dissolution efficiency is controlled by a suite of coordinately expressed core gene modules, and whether these gene modules are conserved within *Priestia* strains and constitute a universal adaptive strategy to a range of silicate and oxide minerals (biotite, potassium-feldspar, olivine, and hematite) environments. Moreover, we attempt to discuss the potential role of genomic plasticity, particularly via plasmid transfer, in driving functional divergence in mineral dissolution among closely related *Priestia* strains. These findings will provide a predictive framework for the geomicrobial contributions of diverse *Priestia* species and valuable insights into the molecular mechanisms governing the mineral dissolution efficiency of mineral-dissolving *Priestia* strains.

## RESULTS

### Divergent biotite dissolution phenotypes between PMA2-2 and PMD1-1

**Fig 1:**
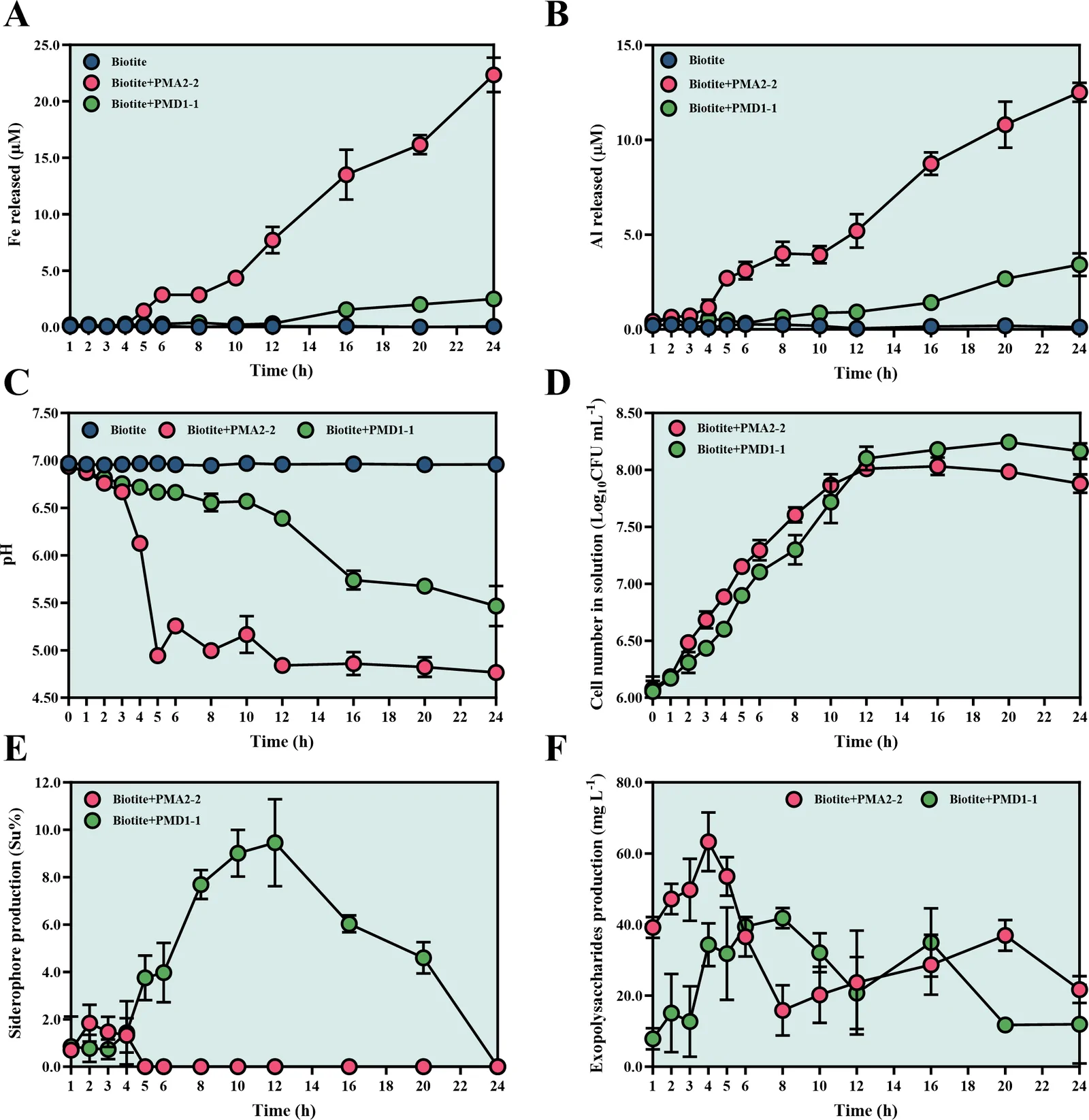
Biotite dissolution experiment of PMA2-2 and PMD1-1. Fe (**A**) and Al (**B**) concentrations, pH value (**C**), cell number (**D**), and siderophore (**E**) and exopolysaccharide (**F**) production in the presence of PMA2-2 and PMD1-1 for 0, 1, 2, 3, 4, 5, 6, 8, 10, 12, 16, 20, and 24 h of incubation. Error bars represent ± one standard deviation (*n* = 3). Two-tailed *P* values for unpaired t tests are indicated between PMA2-2 and PMD1-1.

**Fig 2:**
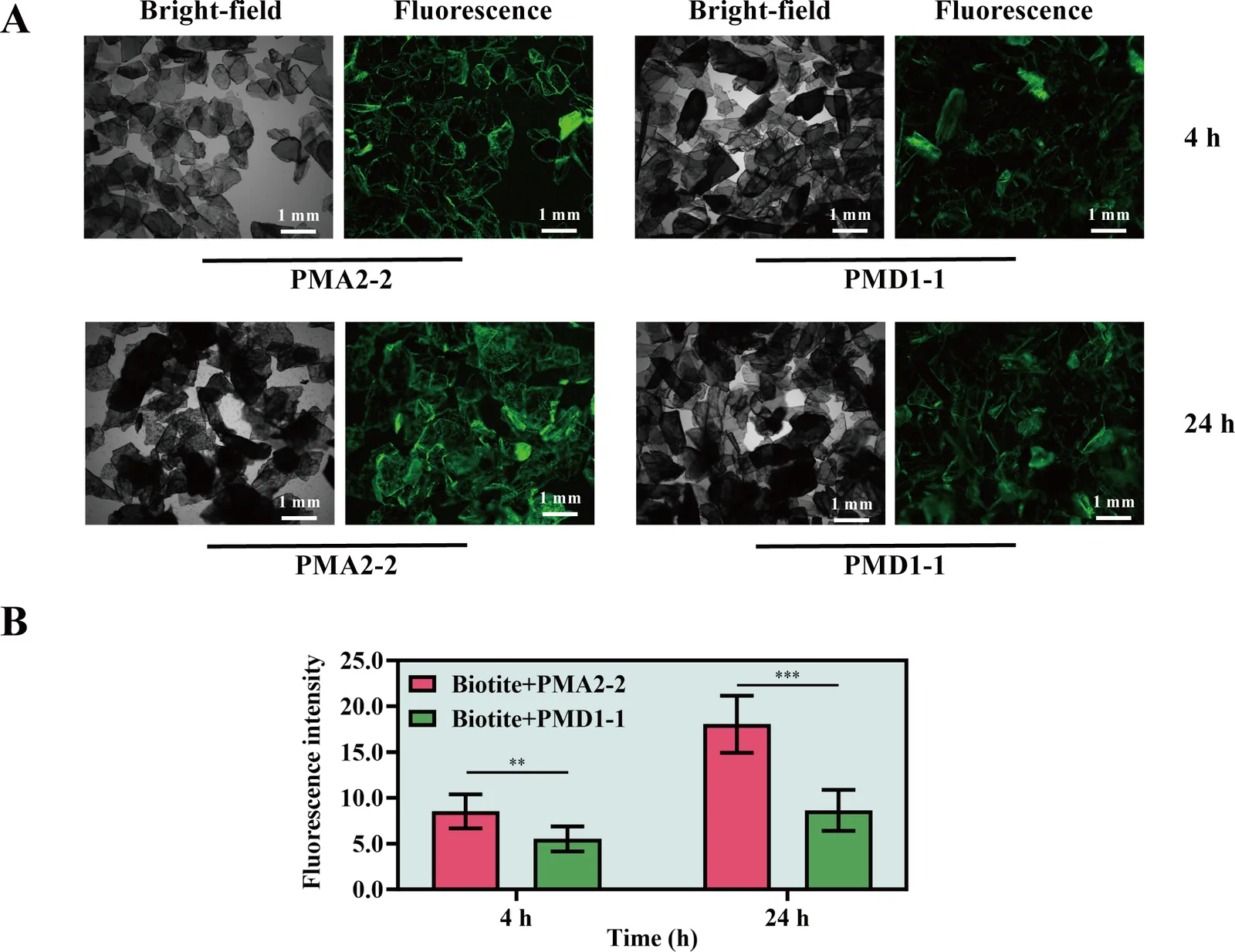
Observation of biofilm formation on the biotite surfaces. Fluorescence microscope images (**A**) and the fluorescence intensity (**B**) of the biofilm formation in the presence of PMA2-2 and PMD1-1 after 4 and 24 h of incubation. Error bars represent ± one standard deviation (*n* = 3). Two-tailed *P* values for unpaired t tests are indicated between PMA2-2 and PMD1-1, **P < 0.01, ***P < 0.001.

Consistent with our hypothesis of divergent functional strategies, PMA2-2 exhibited a significantly more pronounced mineral dissolution phenotype than PMD1-1. PMA2-2 increased dissolved Fe (2.9–22.3 μM) and Al (3.1–12.5 μM) concentrations in the medium by 2.7-fold to 9.3-fold relative to PMD1-1 (Fe, 0.3–2.5 μM and Al, 0.3–3.4 μM) between 6 and 24 h (*P* < 0.01; Fig. 1A and B). Inoculation with PMA2-2 and PMD1-1 significantly (*P* < 0.01) reduced the pH values (ranging from 6.13 to 4.77 in the presence of PMA2-2 and 6.72 to 5.47 in the presence of PMD1-1) in the medium between 4 h and 24 h relative to the control (Fig. 1C). Cell numbers (8.1 log_10_ CFU mL^−1^) in the medium increased steadily for PMD1-1 up to 20 h, while for PMA2-2, they plateaued after 12 h (8.0 log_10_ CFU mL^−1^) (Fig. 1D). The siderophore levels in the medium ranged from 0.8% to 1.8% between 1 and 4 h but were not detected between 4 and 24 h in the presence of PMA2-2, while the siderophore levels in the medium significantly (*P* < 0.05) increased over time (0.8%–9.5%) between 1 and 12 h, then decreased (4.6%–6.0%) between 16 and 20 h (Fig. 1E). PMA2-2 significantly (*P* < 0.05) increased EPS concentrations (ranging from 39.2 mg L^−1^ to 63.3 mg L^−1^) in the medium by 68% to 396% compared with PMD1-1 (EPS concentrations, ranging from 7.9 mg L^−1^ to 34.6 mg L^−1^) between 1 h and 5 h of incubation, while PMA2-2 significantly (*P* < 0.05) increased EPS concentrations (ranging from 21.7 mg L^−1^ to 36.9 mg L^−1^) in the medium by 81% to 215% compared with PMD1-1 (EPS concentrations, ranging from 11.7 mg L^−1^ to 12.0 mg L^−1^) between 20 h and 24 h (Fig. 1F). All bacterial cells in the medium from each independent flask were collected for the determination of cell-associated Fe contents in this study. The cell-associated Fe content in PMA2-2 increased until 12 h and stabilized, whereas in PMD1-1, it increased until 12 h and then declined (Fig. S1). Fluorescence microscopy revealed more robust and developmentally sustained biofilm formation by PMA2-2 on biotite at both 4 and 24 h compared to PMD1-1 (Fig. 2A and B). In the absence of biotite, the pH values in the medium decreased from 6.41 to 4.63 and 6.62 to 5.29 between 4 and 24 h in the presence of PMA2-2 and PMD1-1, respectively (Fig. S2A), while the cell numbers (ranging from 6.1 log_10_ CFU mL^−1^ to 7.8 log_10_ CFU mL^−1^) in the medium increased over time between 4 h and 12 h, then the cell numbers significantly (*P* < 0.05) decreased after 12 h (Fig. S2B). No significant difference in the siderophore production was observed between PMA2-2 and PMD1-1 between 4 h and 12 h, while PMA2-2 produced higher levels of siderophores in the medium than PMD1-1 between 12 h and 24 h in the absence of biotite (Fig. S2C). Moreover, the EPS concentrations in the medium without biotite ranged from 9.1 mg L^−1^ to 46.9 mg L^−1^ in the presence of PMA2-2 and 18.0 mg L^−1^ to 33.8 mg L^−1^ in the presence of PMD1-1 (Fig. S2D). In the acid buffer medium, PMA2-2 increased dissolved Fe (0.8–3.4 μM) concentrations in the medium by 42% to 60% relative to PMD1-1 (Fe, 0.5– 2.4 μM) between 12 and 24 h (*P* < 0.05; Table S1); the pH values in the medium ranged from 6.36 to 6.10 and 6.32 to 6.16, while the siderophore levels in the medium ranged from 26.3% to 46.4% and 34.3% to 57.2% between 12 and 24 h in the presence of PMA2-2 and PMD1-1, respectively (Table S1). Moreover, the cell numbers in the medium ranged from 8.23 to 8.32 log_10_ CFU mL^−1^ and 8.02 to 8.19 log_10_ CFU mL^−1^ in the presence of PMA2-2 and PMD1-1, respectively, in the buffer medium (Table S1). In the presence of PMA2-2 and PMD1-1, significantly (*P* < 0.05) lower dissolved Fe concentrations and higher pH values, siderophore levels, and cell counts were observed in the acid buffer medium than those in the no buffer medium (Fig. 1; Table S1). These results demonstrate that PMA2-2 had a higher ability to promote the dissolution of biotite through enhanced production of acids, EPS, and biofilm formation on the mineral surfaces.

### Genomic basis for phenotypic divergence

The genomic basis for phenotypic divergence was evident in gene content and architecture. PMA2-2 possesses one chromosome (5,119,230 bp) and seven plasmids (746,194 bp), whereas PMD1-1 has one chromosome (3,936,812 bp) and one plasmid (35,653 bp) (Fig. S3 and S4). The whole-genome similarity was 85.47%, indicating phylogenetic proximity at the subspecies level (Fig. S5A). PMA2-2 and PMD1-1 possess 5,945 and 4,016 genes, respectively (Fig. S3 and S4), and the strains share 3,157 core genes, with PMA2-2 and PMD1-1 possessing 2,123 and 748 strain-specific genes, respectively (Fig. S5B). Chromosomal genes related to biofilm formation (*lrgA*), EPS biosynthesis (*pgaB, pgaD,* and *crp*), organic acid metabolism (*fumC, nagK, fahA, aarC, ttuC,* and *gudD*), cell wall component (*vanY*), and flagellar assembly (*ropN*) were present in PMA2-2 but absent in PMD1-1 (Table S2). Notably, genes for EPS biosynthesis (*wecB*), amino acid metabolism (*ansA* and *hisC*), cell wall synthesis (*murF*), two-component systems (*kinE, kinA, yesN, liaR, vanRC, vanSC, phoP,* and *kinB*), and quorum sensing (*nisP* and *nisK*) were located on both the chromosome and plasmids of PMA2-2. Strikingly, key genes for biofilm formation (*bapA*), organic acid metabolism (*fahA*), two-component systems (*saeS* and *devR*), and a complete nisin-like quorum sensing cluster (*nisRCFBA*) were located exclusively on plasmids in PMA2-2 (Table S3). These results demonstrate that the efficient mineral-dissolving PMA2-2 possesses more genes associated with biofilm formation, cell wall/membrane biogenesis, EPS biosynthesis, organic acid metabolism, two-component systems, and quorum sensing in the genome, which are likely responsible for the enhanced mineral dissolution activity of the strain.

### Comparative transcriptional profiling reveals upregulated genes related to mineral dissolution by PMA2-2

**Fig 3:**
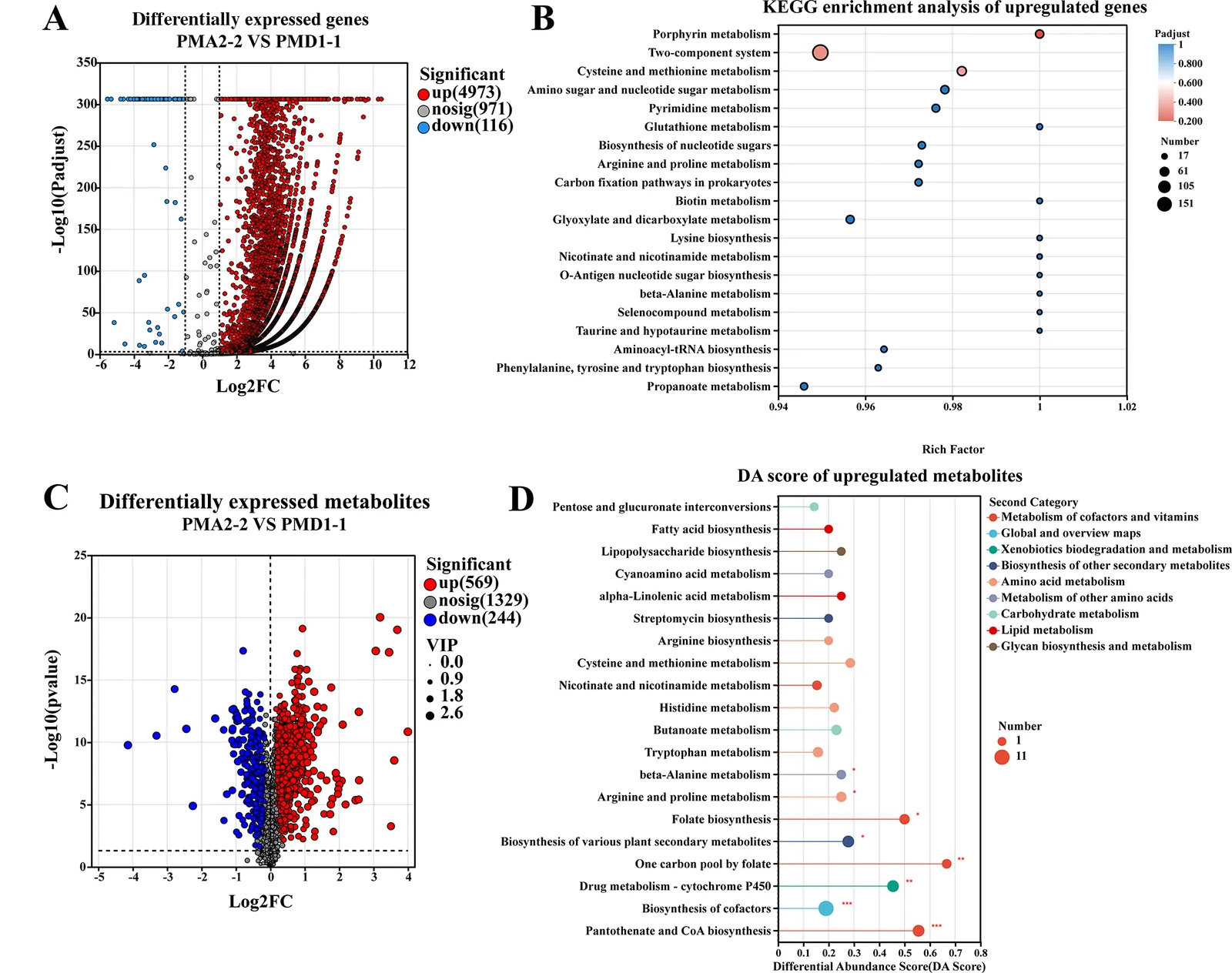
Differentially expressed genes and produced exometabolites between PMA2-2 and PMD1-1. Significantly upregulated and downregulated genes in PMA2-2 compared with those in PMD1-1 (**A**), KEGG enrichment analysis of upregulated genes in PMA2-2 (**B**), significantly upregulated and downregulated produced exometabolites in the presence of PMA2-2 compared with those in the presence of PMD1-1 (**C**), and differential abundance score of upregulated produced exometabolites in the presence of PMA2-2 (**D**).

To further investigate the changes in gene expression between PMA2-2 and PMD1-1 during biotite dissolution and the associated molecular mechanisms, differentially expressed genes (DEGs) exhibiting a fold change greater than 2.0 and a corrected *P-*value below 0.01 were analyzed based on the comparative transcriptomes between PMA2-2 and PMD1-1 cultivated in GMM with biotite. Transcriptomics confirmed the active expression of PMA2-2’s genomic potential, revealing system-wide upregulation of core genes related to mineral dissolution. After 12 h, a comparison between PMA2-2 and PMD1-1 cells grown with biotite identified 4,973 upregulated and 116 downregulated genes in PMA2-2 compared with the genes expressed in PMD1-1 (Fig. 3A). To better understand the molecular mechanisms involved in enhanced biotite dissolution by PMA2-2, we focused on the upregulated DEGs in PMA2-2 compared with those in PMD1-1 in this study. Enrichment analysis of the upregulated DEGs showed significant overrepresentation of pathways linked to biofilm formation, EPS biosynthesis, amino acid metabolism, organic acid metabolism, proton efflux, and flagellar assembly in PMA2-2 (Table 1, Fig. 3B). Genes involved in two-component systems, quorum sensing, secondary messenger signaling, ABC transporters, secretion systems, phosphotransferase systems, and cell wall components were also significantly upregulated in PMA2-2 (Fig. S6). These results reveal more upregulated DEGs and pathways potentially related to mineral dissolution by PMA2-2.

**TABLE 1:** Upregulated mineral dissolution-related differentially expressed genes in PMA2-2 compared with those of PMD1-1^*a*^

Gene ID	Gene name	Description	Function	Log_2_FC (PMA2-2 vs PMD1-1)
Biofilm formation				
pD_gene0068	*bapA*	Tandem-95 repeat protein	Large surface protein	3.8***
gene4504	*spo0A*	Sporulation transcription factor	Sporulation	4.4***
gene1301	*lrgB*	LrgB family protein	Autolysis	5.1***
EPS biosynthesis				
gene0952	*pgaC*	Glycosyltransferase	PGA biosynthesis	5.5***
gene4947	*glgC*	Sugar phosphate nucleotidyltransferase	Glycogen biosynthesis	4.2***
pD_gene0018	*wecB*	UDP-N-acetylglucosamine 2-epimerase	Polysaccharide biosynthesis	4.7***
gene5180	*wecC*	Nucleotide sugar dehydrogenase	Polysaccharide biosynthesis	4.1***
gene0684	*crp*	Crp/Fnr family transcriptional regulator	Regulator	4.9***
Amino acid metabolism				
gene4437	*lysA*	Diaminopimelate decarboxylase	Lysine	5.4***
pB_gene0142	*ansA*	Asparaginase	L-Aspartate	3.3***
gene4839	*ald*	Alanine dehydrogenase	L-Alanine	5.1***
gene4281	*sdaA*	L-serine ammonia-lyase	Serine	3.3***
gene5212	*glyA*	Serine hydroxymethyltransferase	Glycine	3.9***
gene5024	*thrC*	Threonine synthase	Threonine	3.4***
gene5120	*hisD*	Histidinol dehydrogenase	L-Histidine	3.8***
gene0368	*nos*	Nitric oxide synthase oxygenase	Arginine	4.6***
pB_gene0126	*hisC*	Histidinol-phosphate transaminase	Tyrosine	1.2**
gene4708	*pheA*	Prephenate dehydratase	Phenylalanine	3.3***
Organic acid metabolism				
gene3144	*gltA*	Citrate synthase	Citrate	4.6***
gene2639	*acnA*	Aconitate hydratase	cis-Aconitate	3.6***
gene1367	*pyc*	Quinone oxidoreductase	Oxaloacetate	3.7***
gene2388	*fumC*	Class II fumarate hydratase	Fumarate	4.4***
gene2536	*acs*	Acyl-CoA ligase	Acetate	5.6***
gene4821	*icd*	NADP-dependent isocitrate dehydrogenase	Oxalosuccinate	2.8***
gene2020	*ttuC*	Tartrate dehydrogenase	L-Tartrate	6.2***
gene0643	*aceA*	Isocitrate lyase	Glyoxylate	5.6***
gene1983	*gudD*	Glucarate dehydratase	D-Glucarate	6.1***
gene4777	*sdhC*	Succinate dehydrogenase cytochrome b558 subunit	Succinate	3.6***
gene4167	*korA*	Acceptor oxidoreductase subunit alpha	2-Oxo-glutarate	5.2***
gene4820	*mdh*	Malate dehydrogenase	(S)-Malate	3.3***
pB_gene0125	*fahA*	Fumarylacetoacetase	Fumarate	2.4***
Proton efflux				
gene5206	*atpB*	F_0_F_1_ ATP synthase subunit A	F-type ATPase	4.4***
gene5014	*ndh*	NAD(P)/FAD-dependent oxidoreductase	NADH dehydrogenase	4.5***
gene1368	*ctaA*	Heme A synthase	Cytochrome c oxidase	7.9***
Flagellar assembly				
gene5191	*flgF*	Flagellar hook-basal body protein	Basal body/hook	5.4***
gene5165	*flgM*	Flagellar biosynthesis anti-sigma factor	Regulator	4.3***

^*a*^ ***, *P* < 0.001; **, *P* < 0.01.

### Metabolomic signatures of enhanced mineral dissolution by PMA2-2

Comparative metabolomics revealed distinct exometabolite profiles between PMA2-2 and PMD1-1. A total of 322 and 137 differentially produced exometabolites (DEEs) were specific to PMA2-2 and PMD1-1, respectively, with 1,796 common to both (Fig. S7A). PMA2-2 showed 569 significantly upregulated and 244 downregulated DEEs (*P* < 0.05, VIP >1) compared to PMD1-1 (Fig. 3C). To better characterize the enhanced biotite dissolution capacity of PMA2-2 compared with PMD1-1, we focused on the significantly upregulated DEEs in the presence of PMA2-2 (Tables S4 and S5). The significantly upregulated DEEs were primarily enriched in organic acids and derivatives, lipids, organoheterocyclic compounds, and organic oxygen compounds (Fig. S7B). KEGG pathway analysis indicated enrichment in amino acid metabolism, organic acid metabolism, glycan biosynthesis, cofactor metabolism, and lipid metabolism (Fig. 3D). These results demonstrate that more upregulated DEEs are involved in organic acids, lipids, organoheterocyclic compounds, and organic oxygen compounds related to mineral dissolution by PMA2-2.

### Multi-omics integration reveals coordinated gene-metabolite networks

A multi-omics analysis was performed to assess the expression levels of 4,973 upregulated genes and 569 upregulated exometabolites in PMA2-2 compared with those in PMD1-1. In this study, two key exometabolites from each category (EPSs, amino acids, organic acids, and cell wall components, which are related to mineral dissolution) were selected for correlation analysis with relevant genes. Exometabolites related to biofilm/EPS (ADP ribose and isorhamnetin 3-rutinoside-7-glucoside) showed significantly (*P* < 0.05) positive correlations with 19 corresponding biosynthesis genes (*glgA*/*glgB*/*glgC*/*glgP*, *spoOA*/*spoOF*/*spoOB*, *wecB*/*wecC*, *epsA*/*epsB*, *lrgA*/*lrgB*, *crp*, *bapA*, *remA*, *cysE*, *degU*, and *pgaC*) (Fig. S8). Amino acids (Asp-Asp-Tyr and Lys-Glu-Arg-Phe-Ala) significantly (*P* < 0.05) correlated with nine amino acid metabolism-related genes (*sdaA*, *ald*, *proC*, *hisC*, *ansA*, *nos*, *gltB*, *lysA*, and *gltD*). Organic acids (glucose-lactate-acetate and N-acetyl-serylaspartic acid) significantly (*P* < 0.05) correlated with 16 organic acid metabolism-related genes (*sdhB*, *mdh*, *korA*, *korB*, *glgD*, *pyc*, *ttuC*, *aarC*, *aceA*, *nagK*, *glcF*, *glcE*, *fumA*, *gltA*, *acs*, and *gudD*). Cell wall components (ADP ribose and adenosine diphosphate ribose) significantly (*P* < 0.05) correlated with 22 cell wall biosynthesis-related genes (*murB*/*murF*/*murA*/*murC*/*murE*, *pbpA*/*pbpG*/*pbpB*, *mrcA*, *uppS*, *tagA*, *spoVD*, *dgkA*, *ddl*, *bacA*, *wecA*, *mraY*, *ugtP*, *vanY*, *tagT_U_V*, *bcrC*, and *ltaS*) (Fig. S8). These results suggest strong positive correlation between gene expression levels and metabolic activities involved in mineral dissolution by PMA2-2.

### Phylogenetic conservation of key proteins related to mineral dissolution

**Fig 4:**
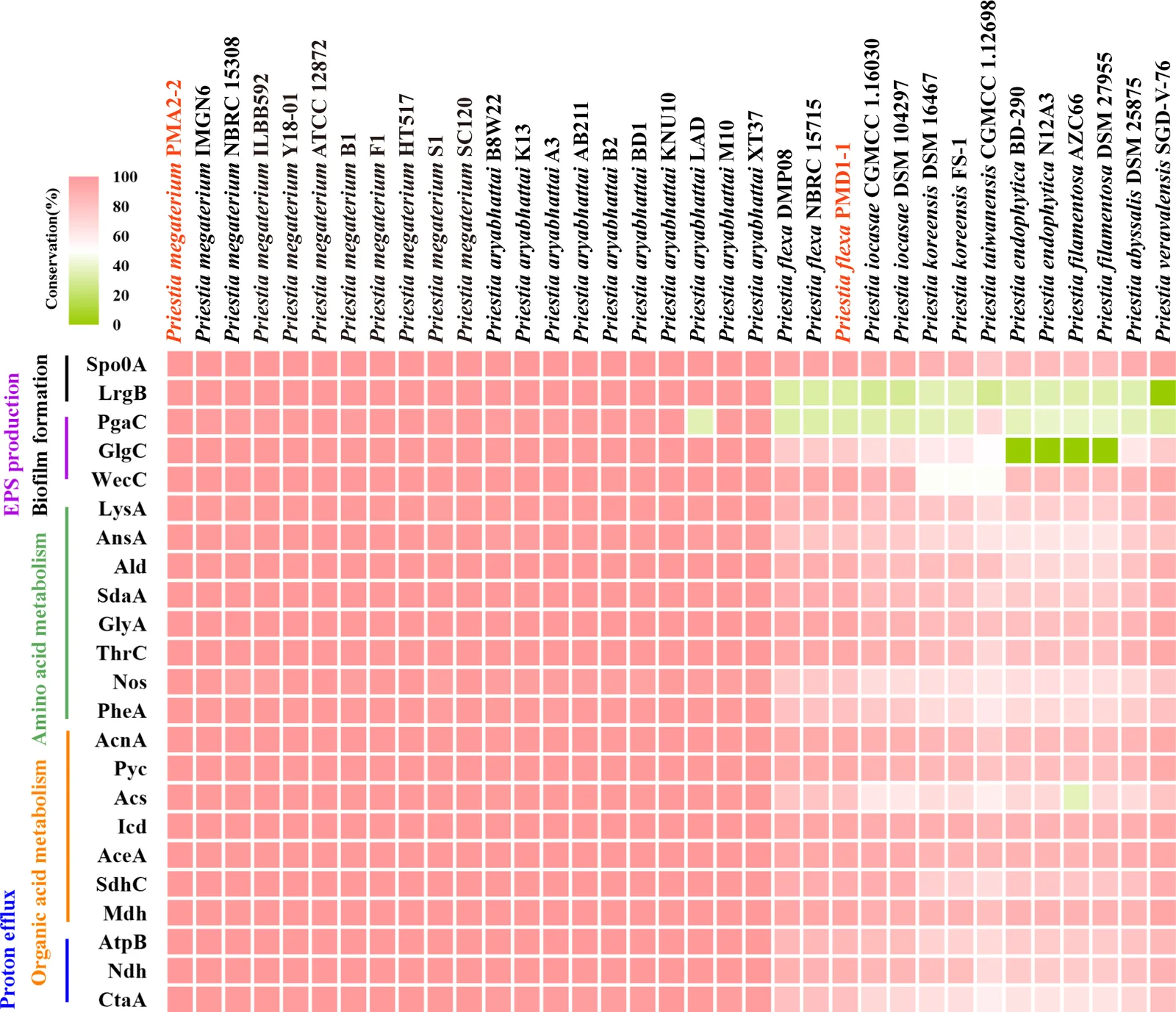
Evolution analysis of proteins related to mineral dissolution by PMA2-2 in *Priestia* strains. To compare the conservation of the mineral dissolution-related proteins among different *Priestia* strains, we blasted the whole-genome of PMA2-2 with 35 different *Priestia* strains. The colors represent percent identity.

To assess universality, we analyzed the conservation of 23 proteins potentially associated with mineral dissolution, corresponding to the most significantly upregulated DEGs, across the genomes of 35 *Priestia* strains (Fig. 4). Proteins were related to biofilm formation (Spo0A and LrgB), EPS production (PgaC, GlgC, and WecC), amino acid metabolism (LysA, AnsA, Ald, SdaA, GlyA, Thrc, Nos, and PheA), organic acid metabolism (AcnA, Pyc, Acs, Icd, AceA, SdhC, and Mdh), and proton efflux (AtpB, Ndh, and CtaA). Homologs of these mineral dissolution-related proteins were identified in 10 *Priestia* species (Fig. 4). Sequence identities of these mineral dissolution-related proteins were high (96%–100%) among 21 *P. megaterium* and *P. aryabhattai* strains. Among 14 strains of other species (*P. flexa, P. iocasae, P. koreensis, P. taiwanensis, P. endophytica, P. filamentosa, P. abyssalis,* and *P. veravalensis*), identities ranged from 48% to 94%, with lower conservation for LrgB, PgaC, and GlgC (Fig. 4). These analyses reveal a core set of highly conserved proteins potentially associated with mineral dissolution across the genus *Priestia*.

### Generalizability of the dissolution response to diverse minerals

**Fig 5:**
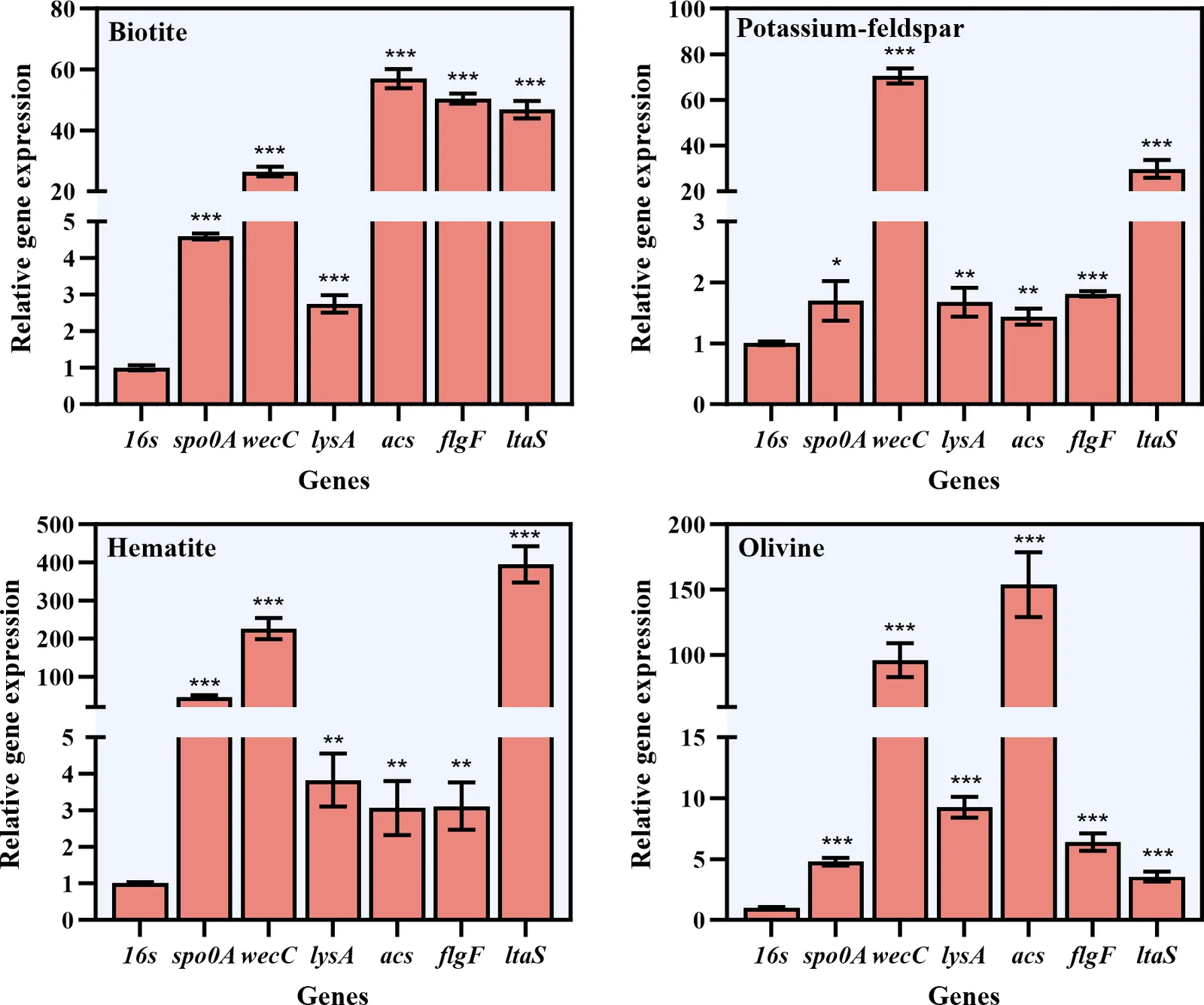
Relative expression of mineral dissolution-related genes between the absence and presence of different minerals. The mineral dissolution-related genes are *spoOA*, *wecC*, *lysA*, *acs*, *flgF*, and *ltaS*, while the minerals include biotite, potassium-feldspar, hematite, and olivine. Error bars represent ± one standard deviation (*n* = 3). Statistical analysis for each time point was performed using an independent two-tailed unpaired t-test between each gene and the 16S rRNA gene. *P*-values are indicated as follows: *P < 0.05, **P < 0.01, ***P < 0.001.

To test the generality of the identified pathways, we examined PMA2-2’s dissolution of other minerals and the associated genetic response. PMA2-2 significantly (*P* < 0.001) increased dissolved Al (13.09–21.48 μM) from potassium feldspar relative to the controls (0.11–0.19 μM Al), increased dissolved Fe (25.95–71.43 μM) from hematite relative to the control (0.04 μM Fe), and increased dissolved Fe (15.24–30.95 μM) and Mg (1.30–1.43 μM) from olivine relative to the controls (0.04–0.05 μM Fe and 0.89 μM Mg) after 12–24 h (Table S6). The cell counts of PMA2-2 ranged from 5.82 to 8.06 log_10_ CFU mL^−1^, and the pH values in the medium ranged from 4.60 to 4.91 in the presence of PMA2-2 and 6.99 to 7.31 in the absence of the strain after 12–24 h (Table S6). To evaluate gene expression response of PMA2-2 to these minerals based on the DEGs identified from the transcriptomic data, six selected upregulated DEGs potentially associated with mineral dissolution, including *spo0A*, *wecC*, *lysA*, *acs*, *lgF*, and *ltaS* encoding sporulation transcription factor, nucleotide sugar dehydrogenase, diaminopimelate decarboxylase, acyl-CoA ligase, flagellar hook-basal body protein, and lipoteichoic acid synthase family protein, respectively, in PMA2-2, were analyzed using RT-qPCR in the absence and presence of these minerals (Fig. 5). The expression of six genes (*spo0A, wecC, lysA, acs, flgF,* and *ltaS*) was significantly induced (1.4- to 395-fold) in PMA2-2 by all structurally diverse minerals, such as biotite, potassium feldspar, olivine, and hematite, which are phyllosilicate, tectosilicate, nesosilicate, and oxide minerals, respectively (Fig. 5). These results demonstrate the important role of PMA2-2 in the dissolution of these structurally diverse minerals and suggest the role of these genes in mineral dissolution by this strain.

## DISCUSSION

In this study, we employed comparative phenotypic and multi-omics analyses to decipher the divergent mineral dissolution phenotypes between the efficient mineral-dissolving strain PMA2-2 and inefficient mineral-dissolving strain PMD1-1, along with the underlying physiological and molecular mechanisms. Our experiments linked PMA2-2’s enhanced biotite dissolution phenotype to the enhanced acidification and EPS production in the medium as well as biofilm formation on the mineral surface (Fig. 1 and 2). The genome of PMA2-2 contains more genes associated with organic acid metabolism, cell wall/membrane components, EPS biosynthesis, and biofilm formation, all of which may contribute to enhanced mineral dissolution by this strain (Table S2). Moreover, comparative transcriptomic and metabolomic analysis revealed that biotite significantly upregulated the expression of genes and production of exometabolites related to organic acid and EPS production, amino acid metabolism, biofilm formation, and cell wall/membrane components in PMA2-2, which may be implicated in biotite dissolution by this strain.

Organic acids, siderophores, and EPS can enhance dissolution by complexing with ions in solution (7, 27, 28). The production of EPS facilitates biofilm formation, thus bringing adsorption sites on the exterior of the cell closer to the mineral surface and generating locally higher concentrations of weathering agents such as siderophores and acids (29, 30). *Priestia strains* enhance silicate mineral dissolution through the production of organic acids, protons, EPS, siderophores, and biofilms (23, 25, 26) via acidolysis, chelation, and adsorption mechanisms. In this study, PMA2-2 exhibited a significantly enhanced mineral dissolution activity compared to PMD1-1. We observed enhanced acidification and increased EPS production and biofilm formation on the biotite surfaces during the mineral dissolution process of PMA2-2 (Fig. 1C and F). Notably, the siderophore production by PMA2-2 in the presence of biotite was detected only in the early stage of the experiment (Fig. 1E), whereas the siderophore production by PMD1-1 was detected throughout the whole mineral dissolution process of PMD1-1 (Fig. 1E). Inoculation with PMA2-2 significantly resulted in reduced acidification and Fe release from biotite and enhanced siderophore production under acid buffer conditions (Table S1) and enhanced acidification and Fe release from the mineral and reduced siderophore production under no acid buffer conditions (Fig. 1), suggesting the important role of the medium acidification on mineral dissolution by PMA2-2. These results suggest that PMA2-2 significantly enhances mineral dissolution through sustained acidification and robust and prolonged biofilm development (Fig. 1C and 2). In contrast, PMD1-1 exhibits a more moderate response with weaker acidification, transient biofilm formation, and a strategy that appears to prioritize siderophore-mediated mineral dissolution (Fig. 1C, E and 2).

The genomic basis of PMA2-2 demonstrated that the key regulatory (*saeS* and *devR* responsible for two-component system and *nisRCTBA* and *comQ/P* responsible for quorum sensing) and structural (*bapA* responsible for biofilm formation and *fahA* responsible for organic acid metabolism) clusters are located only on the plasmids of PMA2-2 (Table S3). Two-component systems, quorum-sensing systems, biofilm formation, and organic acid metabolism have been reported to play an important role in biotite dissolution by bacteria (7, 13, 14, 17, 31). These results strongly suggest that plasmid transfer may have played a critical role in augmenting PMA2-2’s weathering capabilities, fine-tuning traits like biofilm integrity and social coordination. Genomic analysis suggests that the expanded suite of plasmid-borne genes related to mineral dissolution likely underlies the efficient weathering phenotype of PMA2-2 (Fig. 1; Tables S2 and S3). However, the molecular mechanisms employed by gram-positive *Priestia* strains in dissolving silicate minerals are poorly characterized. Wu et al. (25) reported that the strong potassium feldspar *dissolution by the* efficient mineral-dissolving strain *Priestia megaterium* NK851 was not attributed to the acidic pH but was attributed to the extracellular potassium feldspar-binding proteins. Transcriptomic analysis revealed potassium feldspar-induced upregulation of genes related to pyruvate metabolism, two-component systems, DNA repair, and oxidative stress pathways in the mineral-dissolving strain *Priestia aryabhattai* SK1-7 (26). Furthermore, Sheng et al. (23) reported biotite-induced upregulation of genes related to glyoxylate and dicarboxylate metabolism, amino acid biosynthesis, the tricarboxylic acid cycle, and ABC transporters in the mineral-dissolving strain *Priestia aryabhattai* C4-10. In this study, transcriptomics confirmed the active expression of PMA2-2’s genomic potential, revealing system-wide upregulation of core dissolution genes involved in organic acid and EPS production, proton efflux, biofilm formation, amino metabolism, flagellar assembly, and environmental sensing (including two-component systems and quorum sensing) as well as nutrient transport systems (ABC transporters) (Table 1, Fig. 3A). These systems may regulate the production of acidic exometabolites and surface adsorption as well as biofilm formation during the mineral dissolution process of PMA2-2 (17, 31). Gene *spo0A* encodes the master regulator Spo0A to coordinate biofilm formation and metabolic shifts in bacteria (21, 22). The significant upregulation of *spo0A* may be associated with biofilm formation and mineral dissolution by PMA2-2 (Fig. 1 and 2). Future research should focus on experimentally validating the regulatory role of Spo0A to confirm its direct causal role in enhancing mineral dissolution. Our previous work has shown that the quorum-sensing TraI/TraR system contributes to biotite dissolution, EPS production, and cell adsorption and biofilm formation on the mineral surface (31). The cooperative upregulation of the plasmid-borne nisin-like quorum-sensing cluster (*nisRK*) and the *comQP* system suggests that cell density-dependent signaling regulates the transition from cell attachment to mature biofilm formation (32) and may be involved in mineral dissolution by PMA2-2. Furthermore, several genes involved in TCSs (e.g., *phoPR* for phosphate sensing and *liaRS* for cell envelope stress) were upregulated, likely detecting nutrient limitation and surface contact signals in mineral microenvironments (33). TCS ResS/ResR has been reported to play an important role in biotite dissolution by bacteria (17). The convergence of these regulatory inputs—developmental (Spo0A), communal (quorum sensing), and environmental (TCSs)—onto shared effector pathways may provide a robust, responsive genetic framework that allows PMA2-2 to dynamically optimize its mineral dissolution strategy. The upregulation of genes such as *ltaS* (encoding lipoteichoic acid synthase) and *bapA* (encoding biofilm-associated protein) in PMA2-2 may provide a molecular basis for its enhanced attachment to mineral surfaces. Increased lipoteichoic acid synthesis likely alters cell surface charge and hydrophobicity, enhancing initial adhesion to mineral surfaces (34–36). Concurrently, upregulation of the plasmid-borne *bapA* gene may correlate with the enhanced biofilm formation (Fig. 2), likely providing a robust basis for mineral surface adsorption (37). The phenotypic and genomic divergence between PMA2-2 and PMD1-1, isolated from the same niche, potentially underscores the ecological relevance of efficient mineral dissolution. Evolutionarily, the plasmid localization of key accessory genes (such as *bapA* and *nisRCTBA*) in PMA2-2 strongly implies that plasmid transfer may augment core genes related to mineral dissolution. This combination of a conserved core and mobile accessories may represent a potent mechanism for niche specialization in mineral dissolution effectiveness within *Priestia* strains. Furthermore, the significantly upregulated DEEs included the exometabolites linked to amino acids, organic acids, EPS, and cell wall components (Tables S4 and S5), suggesting that PMA2-2 may employ a more active multi-mechanistic strategy (acidolysis, chelation, and adsorption) via concerted exometabolite production. Integration of transcriptomic and metabolomic data revealed correlations between the upregulated genes and produced exometabolites in PMA2-2 (Fig. S8). This strong positive correlation network may provide robust evidence of tightly coupled transcription and metabolite production in PMA2-2, thereby underpinning the efficient execution of its multi-component dissolution strategy. This systems-level coordination between gene expression and corresponding metabolic production may ensure efficient mineral dissolution by PMA2-2.

To assess universality, we analyzed the conservation of 23 proteins related to mineral dissolution, corresponding to the most significantly upregulated DEGs, across the genomes of 35 *Priestia* strains (Fig. 4). This reveals a core set of highly conserved proteins related to mineral dissolution across the genus, supporting the existence of a generalized genetic foundation for mineral dissolution in *Priestia*. The widespread presence of these proteins among known mineral-solubilizing *Priestia* species, including *P. megaterium*, *P. aryabhattai*, *P. flexa*, *P. koreensis*, *P. endophytica*, and *P. filamentosa* (38–41), suggests their conserved role in mineral dissolution across diverse environments. To test the generality of the identified pathways, we examined PMA2-2’s dissolution of other minerals and the associated genetic response (Fig. 5). Notably, we identified a conserved set of core genes (*spoOA*, *wecC*, *lysA*, *acs*, *flgF*, and *ltaS*) in PMA2-2, which were significantly upregulated in the presence of silicate and oxide minerals (Fig. 5). The upregulated genes are not only involved in organic acid metabolism (e.g., *acs*) but also encompass functions related to cell wall synthesis (*ltaS*) and flagellar assembly (*flgF*), polysaccharide biosynthesis (*wecC*), biofilm formation (*spoOA*), and amino acid metabolism (*lysA*) (Table 1; Fig. S6D). Biofilm formation enriches cells at the mineral interface and may directly promote mineral dissolution by maintaining a local microenvironment with elevated concentrations of protons and chelators (14, 42). These results suggest that the core genes related to mineral dissolution are conserved across the genomes of *Priestia* strains (including known mineral-solubilizing *Priestia* species), and these genes are inducible responses to various minerals. The expression of *spo0A, wecC, lysA, acs, flgF, and ltaS* was significantly induced in PMA2-2 during the bacterial dissolution of biotite, potassium feldspar, olivine, and hematite, suggesting the important role of these genes in the dissolution of these minerals by PMA2-2. The direct functional roles of these genes in mineral dissolution await further validation in future work.

Ultimately, our findings may have potential applications for plant nutrients and carbon sequestration. Improved understanding of the underlying molecular mechanisms of silicate dissolution by bacteria contributes to developing microbe-enhanced technologies for managing plant nutrients (43) and strategies for carbon dioxide sequestration (44). The efficient dissolution of two structurally distinct magnesium-bearing silicate minerals (biotite and olivine) by PMA2-2 indicates its potentially broad-spectrum mineral dissolution capability, presenting a novel candidate strain for using gram-positive bacteria to release nutrients from soil primary minerals or enhance carbon sequestration in soil environments.

**Fig 6:**
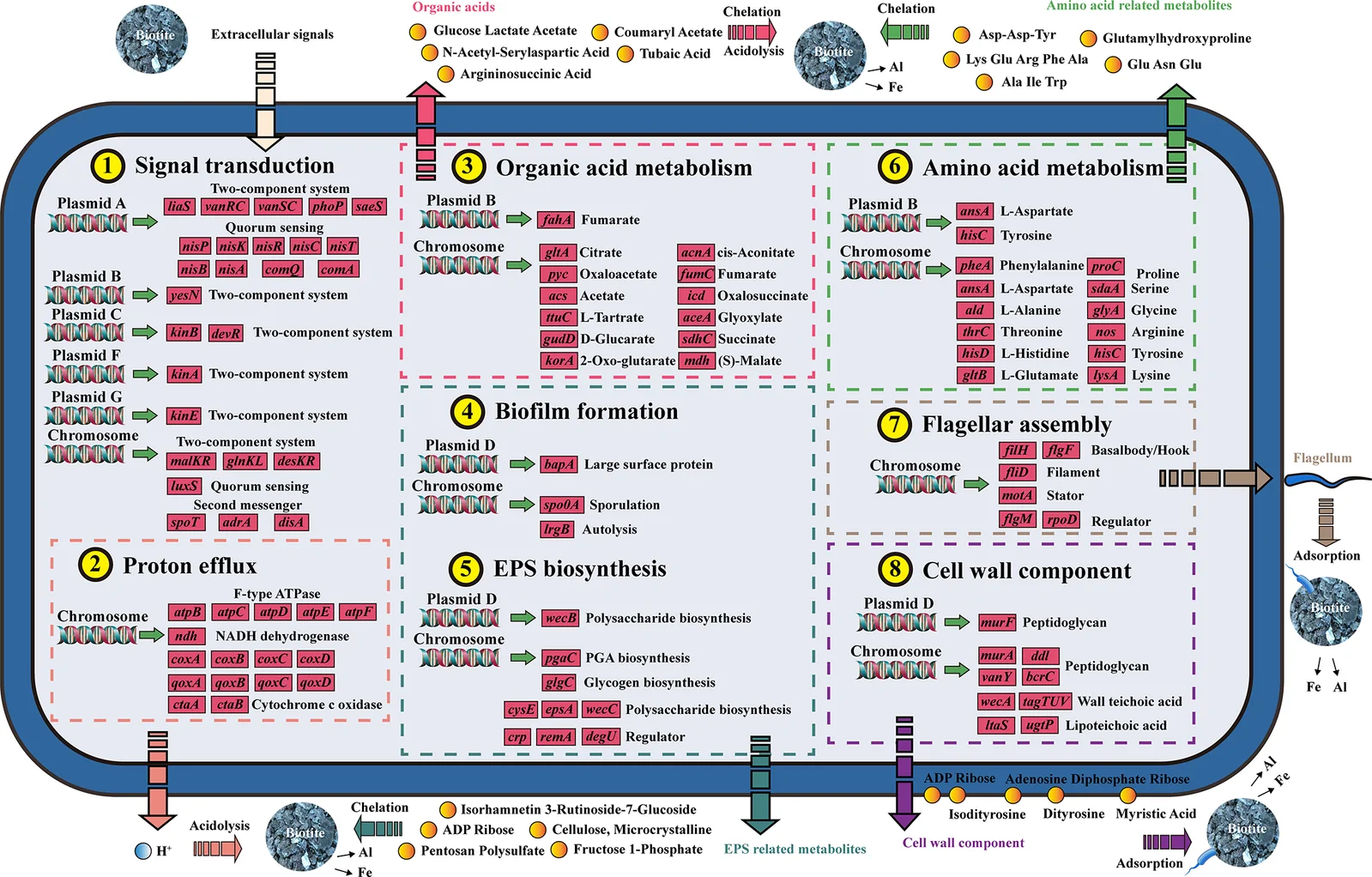
Overview of the molecular mechanisms involved in biotite dissolution by the efficient mineral-dissolving *Priestia megaterium* strain PMA2-2**.** The figure presents an integrative view of the different molecular mechanisms adopted by PMA2-2 in mineral dissolution through physiological, genomic, transcriptomic, and metabolomic approaches. When exposed to extracellular mineral signals, PMA2-2 initiates distinct but coordinated pathways encoded across both its chromosomes and plasmids. (1) Signal transduction relies on two-component systems and quorum-sensing factors, with relevant genes distributed on plasmids A, B, C, F, G, and the chromosome. (2) Proton efflux pathways—driven by chromosomally encoded F-type ATPase, NADH dehydrogenase, and cytochrome c oxidase genes—actively export H^+^ to acidify the local microenvironment. (3) Organic acid metabolism genes on plasmid B and the chromosome generate key organic acids that drive mineral chelation and acidolysis, directly promoting mineral dissolution. (4 and 5) Biofilm formation and exopolysaccharide biosynthesis genes on plasmid D and the chromosome enhance bacterial adhesion to mineral surfaces and provide additional chelating agents. (6) Amino acid metabolism genes on plasmid B and the chromosome produce carboxyl-rich amino acids and their derivatives, which facilitate interfacial mineral interactions through complexation with metal ions. (7 and 8) Flagellar assembly and cell wall remodeling genes on the chromosome and plasmid D further reinforce bacterial colonization on mineral surfaces. The partitioning of these functional genes across genomic compartments underscores a synergistic strategy, where metabolic output, structural adaptation, and regulatory sensing collectively mediate efficient mineral dissolution.

In conclusion, this study reveals that PMA2-2 possesses a greater number of chromosome- and plasmid-borne genes potentially involved in mineral dissolution, and may provide new insights into the coordinated upregulation of multiple genes and exometabolites linking to the enhanced biotite dissolution phenotype of PMA2-2 (Fig. 6). These upregulated genes and exometabolites are involved in organic acid and EPS production, proton efflux, biofilm formation, cell wall components, and flagellar assembly as well as environmental sensing (including two-component systems and quorum sensing) and nutrient transport systems (ABC transporters) (Fig. 6). The genes responsible for efficient mineral dissolution are widespread and phylogenetically conserved across the *Priestia* genus. Furthermore, a range of silicate and oxide minerals can induce the expression of these core genes related to mineral dissolution in the efficient mineral-dissolving *Priestia megaterium* PMA2-2. Our findings not only deepen the understanding of the functional divergence in mineral dissolution and their molecular drivers in gram-positive bacteria but may also provide a theoretical foundation for enhancing the environmental application potential of such strains.

## MATERIALS AND METHODS

### Bacterial strains, minerals, and media

The strains *Priestia megaterium* PMA2-2 (GenBank: CM125967.1) and *P. flexa* PMD1-1 (GenBank: CM125967.1) were isolated from the rhizosphere soil of *Dracaena cochinchinensis* in Purple Mountain, Nanjing, China. Preliminary assays showed that PMA2-2 increased dissolved Fe (16.4 μM) and Al (7.6 μM) concentrations in biotite-amended medium by 1.7- to 6.8-fold compared to PMD1-1 (Fe, 2.1 μM and Al, 2.8 μM) after 24 h of incubation. Phylogenetic analysis placed PMA2-2 and PMD1-1 within the same evolutionary branch of the *Priestia* genus (Fig. S9). The compositions of biotite, potassium-feldspar, olivine, and hematite are provided in Table S7. Minerals were sieved to a controlled particle size of 75–150 μm. Glucose minimal medium (GMM) contained the following: 0.015% MgSO₄·7H₂O, 0.05% Na₂HPO₄·2H₂O, 0.025% (NH_4_)_2_SO₄, 0.01% KNO₃, 0.5% glucose, and pH 7.0. Acid buffer medium contained the following: 0.015% MgSO_4_·7H_2_O, 0.2% Na_2_HPO_4_·2H_2_O, 0.1% NaH_2_PO_4_·2H_2_O, 0.025% (NH_4_)_2_SO_4_, 0.01% KNO_3_, 0.5% glucose, and pH 7.0. Luria–Bertani (LB) medium contained 0.5% yeast extract, 1% tryptone, and 1% NaCl. All chemicals were analytical grade.

### Biotite dissolution experiment

Bacterial biotite dissolution was assessed in triplicate following a modified protocol from Wang et al. (10). Strains were pre-cultured in 3 mL LB medium at 37°C, 160 rpm for 12 h. Cells were harvested by centrifugation, washed, and resuspended in physiological saline (0.85% NaCl solution). Triplicate 220 mL flasks containing 60 mL of sterilized GMM (containing 0.60 g of 75–150 μm particles of biotite) were each inoculated with 0.2 mL of bacterial suspension (10^8^ cells mL^−1^) and incubated at 37°C, 160 rpm. In the study, we prepared individual flasks for each time point. At designated times, culture medium (10 mL) was collected, centrifuged, and filtered (0.45 μm Polyethersulfone). Dissolved Fe and Al concentrations were quantified via inductively coupled plasma optical emission spectrometry (ICP-OES) (Optima 2100 DV, Perkin Elmer, USA). pH was measured from a separate 5 mL filtrate. Cell counts were determined by serial dilution plating (38). EPS from the pellet was quantified using the phenol-sulfuric acid assay (31). Siderophore production was measured as siderophore units (%) via the chromeazurol S (CAS) liquid assay (45). For the determination of cell-associated Fe contents (including cell surface-adsorbed and intracellular accumulated Fe contents), all bacterial cells in the medium were collected from each independent flask by centrifugation (at 8,000 × *g* for 5 min at 4°C). The cells were lyophilized and weighed using an analytical balance (precision 0.1 mg). The lyophilized cells were then digested in 30% hydrogen peroxide (2 h, boiling water bath), filtered, and the Fe concentrations in the filtrate were analyzed by ICP-OES. Furthermore, the pH values, cell numbers, siderophore production, and EPS concentrations in the PMA2-2 or PMD1-1-inoculated medium without biotite were also determined in this study. Meanwhile, acid buffer medium (GBM) was used to evaluate the impacts of acid buffer conditions on Fe release from biotite, cell growth, and siderophore production of PMA2-2 and PMD1-1 in this study. The determination of dissolved Fe concentrations, pH, cell counts, and siderophore production was performed as described above.

### Complete genome sequencing

Genomic DNA from PMA2-2 and PMD1-1 was extracted and purified (46). Sequencing was performed by Majorbio (Shanghai, China) using Illumina HiSeq and PacBio platforms. Raw reads were processed to remove adapters, poly-N sequences, and low-quality sequences. High-quality reads were assembled using SOAPdenovo v2.04. Annotation was performed via the NCBI Prokaryotic Genome Annotation Pipeline (47), with functional classification against the Clusters of Orthologous Groups database (48).

### Transcriptome sequencing

Two group experiments (PMA2-2 and PMD1-1) were established to conduct transcriptome analysis. GMM with biotite was employed for culturing PMA2-2 and PMD1-1 to investigate differential gene expression. Triplicate cultures of PMA2-2 and PMD1-1 were grown in 60 mL GMM with 0.60 g biotite at 37°C. Cells were harvested at the early exponential phase (12 h) for RNA extraction. Total RNA extraction, library construction, and sequencing (150-bp paired-end reads, ~20 million reads/sample) on an Illumina NovaSeq X (Illumina, USA) Plus platform were conducted by Majorbio. rRNA was removed using the Ribo-Zero Magnetic kit (Epicenter, USA). RNA quality was assessed (RIN > 8.0). Clean reads were mapped to the reference genomes of PMA2-2 (GenBank: CM125967.1) and PMD1-1 (GenBank: CM125968.1) using Bowtie2. DEGs were identified with a threshold of fold change > 2 and *P* value < 0.001 using DESeq. The sequences were annotated according to the Gene Ontology (GO), Kyoto Encyclopedia of Genes and Genomes (KEGG), and NCBI protein nonredundant (NR) databases to identify functional genes, and metabolic pathway analyses were conducted. Detailed methodology is in the supplemental material.

### Metabolomic analysis

Metabolomic profiling was performed in six replicates, modified from Xiang et al. (16). Strains were cultured in 60 mL GMM with biotite (0.60 g) at 37°C for 12 h. Cell-free supernatants were collected, and metabolites were extracted using a methanol:water (4:1, vol/vol) solution. Analysis was performed via liquid chromatography-tandem mass spectrometry (LC-MS/MS). Metabolites were identified by matching MS/MS spectra to the HMDB and Metlin databases. Extracellular metabolites with *P* value < 0.05 and a variable importance in projection (VIP) score > 1 were considered significantly differential. Detailed methods are in the supplemental material.

### Biofilm visualization on biotite

Biofilms formed by PMA2-2 and PMD1-1 on biotite surfaces after 4- and 24-h incubation were visualized as described by Sheng et al. (31). Mineral particles were washed thrice with saline to remove unattached cells, stained with SYTO9 nucleic acid stain (ThermoFisher Scientific, USA), rinsed, air-dried, and observed using fluorescence microscopy (Axiovert-100 MBP, Zeiss, Germany).

### Dissolution of other minerals and gene expression analysis

Dissolution of potassium-feldspar, olivine, and hematite by PMA2-2 was assessed in triplicate as described above. Dissolved Al/Fe/Mg, pH, and cell counts were measured after 12 and 24 h. To assess gene expression in response to different minerals, PMA2-2 was cultured in 60 mL of GMM alone or supplemented with 0.6 g biotite, potassium-feldspar, hematite, or olivine, respectively (the particle size of the minerals is 75–150 μm). After 12 h, bacterial cells were collected by centrifugation at 9,200 × *g* for 5 min at 4°C. Total RNA of PMA2-2 was extracted (Bacteria RNA Extraction Kit, Vazyme, China). cDNA was synthesized (Evo M-MLV RT Mix Kit, Accurate Biotechnology, China). RT-qPCR was performed (Applied Biosystems 7500 system, SuperStar SYBR Master Mix, Cwbio, China) with gene-specific primers (Table 2). The 16S rRNA gene served as the reference. The amplification procedures of the genes, including the 16S rRNA gene (approximately 100 bp), included 40 cycles of 95°C for 5 s; 55°C for 15 s and 72°C for 30 s. The 2^−ΔΔCT^ method was used to calculate the relative expression levels.

**TABLE 2:** RT-qPCR primers used in this study

Primer	Sequence (5′−3′)	Primer	Sequence (5′−3′)
16S-QF	AGGTCAAGCCAATCCCATAAA	acs-QF	GGAGATATTGCCGTTCGTAGAG
16S-QR	CATGCTGATCCGCGATTACTA	acs-QR	CGCGGTCACCTGTTAAATAGTA
spo0A-QF	AGCTGCTTGGGTCGATTAC	flgF-QF	TGTGGTACAAAGCCCTGATAAT
spo0A-QR	CTTCAATGGCATGACGAATGG	flgF-QR	ATAAGCTCCGGTAGACGTAGT
wecC-QF	ATGCGTGGGAAGCGATTAAG	ltaS-QF	CCACGATCAGGTAGTAGCATTT
wecC-QR	CCAAGGGTCAACAGCAATACA	ltaS-QR	CGTCTGTTGCTGTGATTCTTTC
lysA-QF	GTACAGGCGCATACGGTTATT		
lysA-QR	GCTGTGCTTCTCCATCTTCTAC		

### Phylogenetic and sequence conservation analyses

16S rRNA sequences of type strains were obtained from ezbiocloud (https://www.ezbiocloud.net/). A phylogenetic tree was constructed in Geneious Prime V2025.0.2 (global alignment, neighbor-joining). Whole genomes of *Priestia* strains were obtained from NCBI, annotated via the SEED server (http://theseed.org), and analyzed for orthologs using RAST (49). Sequences of 23 proteins (corresponding to the most significantly upregulated DEGs involved in EPS production, biofilm formation, amino acid metabolism, organic acid metabolism, and proton efflux in PMA2-2, which are related to mineral dissolution by bacteria) were aligned via the SEED viewer, and a conservation heatmap was generated using TBtools II v2.136. 23

### Statistical analysis

Data of Fe, Al, and Mg and EPS concentrations, pH value, cell number, siderophore production, fluorescence intensity, intracellular Fe content, and relative gene expression were analyzed with GraphPad Prism 9.3.1. Significance was assessed using two-tailed unpaired *t*-tests. For multiple comparisons, appropriate corrections (e.g., Tukey’s test) were applied. Spearman correlation analysis evaluated associations between upregulated genes and metabolites. Network interactions were visualized with Gephi 0.10.1. For transcriptomic analysis, Benjamini-Hochberg multiple testing correction was applied to control the false discovery rate. For metabolomic data, we used two-tailed unpaired t-tests combined with Orthogonal Projections to Latent Structures-Discriminant Analysis (OPLS-DA) to avoid false positives.

## Data Availability

Data generated or analyzed during this study are included in this published article and its supplemental material. The whole genomes of PMA2-2 and PMD1-1 were deposited in the NCBI GenBank database under the accession numbers CM125967 and CM125968, respectively. The original RNA-seq data were deposited in the NCBI SRA database under the accession numbers SRR35022076 and SRR35022077. The original data from the metabolic analysis were deposited in Mendeley Data under the DOI 10.17632/zbgt7979xk.1.

## SUPPLEMENTAL MATERIAL

Supplemental material aem.01201-26-s0001.pdf Supplemental methods, Tables S1 to S7, and Fig. S1 to S9.

ASM does not own the copyrights to Supplemental Material that may be linked to, or accessed through, an article. The authors have granted ASM a non-exclusive, world-wide license to publish the Supplemental Material files. Please contact the corresponding author directly for reuse.
