# Supplementary material for: Genomic traits, transcriptional responses, and metabolic activities determine functional divergence in mineral dissolution between Priestia megaterium PMA2-2 and Priestia flexa PMD1-1
Source: Appl Environ Microbiol. 2026 Aug 10;92(9):e01201-26. doi: 10.1128/aem.01201-26 (PMC13599522; doi:10.1128/aem.01201-26)
Supplement: Supplementary file 1 [file aem.01201-26-s0001.pdf]

Genomic Traits, Transcriptional Responses and Metabolic Activities Determine Functional Divergence in Mineral Dissolution between *Priestia megaterium* PMA2-2 and *Priestia flexa* PMD1-1

Wen Dong, Shengzhi Zhang, Duo Yu, Qi Sheng, Linyan He, Xiafang Sheng

College of Life Sciences, Nanjing Agricultural University, Nanjing 210095, China

Supporting Materials and methods

RNA-seq

Two group experiments (PMA2-2 and PMD1-1) were established to conduct transcriptome analysis. GMM with biotite was used to culture PMA2-2 and PMD1-1 to study differential gene expression in this study. PMA2-2 and PMD1-1 were cultivated in liquid LB medium, respectively, harvested by centrifugation, and washed, and the cell pellets were then resuspended in sterile distilled water. Triplicate 220-ml flasks containing 60 ml of sterilized GMM with 0.60 g of biotite were each inoculated with 0.2 mL of bacterial suspension (10^8 cells mL^{-1}). The flasks were incubated at 37°C. The culture medium was collected at the early exponential growth phase of these strains (12 h of incubation) for bacterial RNA extraction. Total RNA extraction and sequencing were conducted by Majorbio Bio-Pharm Technology Co., Ltd. (Shanghai, China). Total RNA was extracted from the bacteria cells according to the manufacturer's instructions of TRIzol (Invitrogen, USA), and genomic DNA was removed using DNase I (TaKaRa, Japan). rRNA was removed using the Ribo-Zero Magnetic kit (Epicentre, USA). The mRNA was randomly fragmented into short fragments of approximately 200 bp. Using the fragmented mRNA as templates, double-stranded cDNA was synthesized via reverse transcription with random primers (Illumina, USA) and the SuperScript double-stranded cDNA synthesis kit (Invitrogen, USA). Libraries were constructed and sequenced on an Illumina NovaSeq X Plus (Illumina, USA) platform, generating 150-bp paired-end reads with an average sequencing depth of 20 million reads per sample. Clean reads were mapped to the reference genomes of PMA2-2 and PMD1-1 using Bowtie2. We identified genes with a fold change > 2 and p value < 0.001 in a comparison as significant differentially expressed genes (DEGs). RNA-seq transcriptome libraries were constructed using the Illumina TruSeqTM RNA Sample Preparation Kit (San Diego, CA). The sequences were annotated according to the Gene Ontology (GO), Kyoto Encyclopedia of Genes and Genomes (KEGG), and NCBI protein nonredundant (NR) databases to identify functional genes and metabolic pathway analyses were conducted. The raw counts between different samples were normalized based on the TMM method, and the differential expression between groups was analyzed by DEGseq software package.

Metabolomic analysis

Subsequently, 20 mL of bacterial suspension was collected and centrifuged at 12,000 × g for 10 min at room temperature. From the supernatant, 10 mL was transferred to a 50 mL centrifuge tube and freeze-dried using a freeze dryer (LGJ-10, Songyuan Huaxing, Beijing, China). Extracellular metabolites were extracted using 400 μL of a methanol:water (4:1, v/v) solution containing 0.02 mg mL^{-1} of the internal standard L-2-chlorophenylalanine. The samples were homogenized using a frozen tissue grinder (Wonbio-96c, Shanghai Wanbo Biotechnology Co., Ltd) for 6 min at -10 °C and 50 Hz, followed by low-temperature ultrasonic extraction for 30 min at 5 °C and 40 kHz. After storage at -20 °C for 30 min, the samples were centrifuged at 13,000 × g for 15 min at 4 °C. The resulting supernatant was transferred to an injection vial for LC-MS/MS analysis at Majorbio Biopharm Technology Co., Ltd.

LC-MS/MS analysis was performed using a Thermo UHPLC-Q Exactive HF-X system equipped with an ACQUITY HSS T3 column (100 mm × 2.1 mm i.d., 1.8 μm; Waters, USA). The mobile phase consisted of solvent A (0.1% formic acid in water:acetonitrile, 95:5, v/v) and solvent B (0.1% formic acid in acetonitrile:isopropanol:water, 47.5:47.5:5, v/v/v). Mass spectrometry data were acquired using a Thermo UHPLC-Q Exactive HF-X Mass Spectrometer with an electrospray ionization source operating in both positive and negative modes. Raw data were processed using Progenesis QI (Waters Corporation, Milford, USA). Extracellular metabolites were annotated based on the KEGG database for metabolic pathway analysis and compound classification.

Supporting Tables

Table S1. Fe concentrations, siderophore production, pH value, and cell number in the medium supplemented with biotite in the presence of PMA2-2 and PMD1-1 for 12 and 24 h of incubation under buffer conditions

Strain	Time (h)	Dissolved Fe (μM)	Siderophore production (Su%)	pH	Cell number (Log_{10}CFU mL^{-1})
No bacteria	12	0.4 ± 0.4	(-)	6.86 ± 0.01	(-)
	24	0.2 ± 0.2	(-)	6.85 ± 0.01	(-)
PMA2-2	12	0.8 ± 0.2	26.3 ± 4.9	6.36 ± 0.05	8.32 ± 0.03
	24	3.4 ± 0.4	46.4 ± 2.8	6.10 ± 0.06	8.23 ± 0.01
PMD1-1	12	0.5 ± 0.2	34.3 ± 2.1	6.16 ± 0.05	8.19 ± 0.02
	24	2.4 ± 0.4	57.2 ± 0.2	6.32 ± 0.05	8.02 ± 0.02

Note: the values were shown as mean ± SD (n = 3), (-) means not measured. The initial pH values in the presence of PMA2-2 and PMD1-1 were 6.85 ± 0.03 and 6.85 ± 0.01, respectively. The initial cell numbers (Log_{10}CFU mL^{-1}) of PMA2-2 and PMD1-1 were 6.37 ± 0.09 and 6.39 ± 0.09, respectively.

Table S2. The copy number and location of genes related to mineral dissolution of PMA2-2 and PMD1-1

Functions	Genes	Copies		Location	
		PMA2-2	PMD1-1	PMA2-2	PMD1-1
**Biofilm formation**					
Large surface protein	*bapA*	1	1	pD	C
	spo0F	1	1	C	C
Sporulation	*spo0B*	1	1	C	C
	spo0A	1	1	C	C
Autolysis	*lrgA*	1	0	C	(-)
	lrgB	3	1	C	C
**EPS biosynthesis**					
	pgaB	2	0	C	(-)
Poly-N-acetyl-glucosamine (PGA) biosynthesis	*pgaC*	2	1	C	C
	pgaD	1	0	C	(-)
	glgC	2	2	C	C
Glycogen biosynthesis	*glgA*		1	C	C
	glgP		1	C	C
	cysE	2	2	C	C
	wecB	2	2	C(1)/pD(1)	C
Polysaccharide biosynthesis	*wecC*	1	1	C	C
	epsA	1	2	C	C
	epsB	1	2	C	C
	crp	3	0	C	(-)
Regulator	*remA*	1	1	C	C
	degU	1	2	C	C
**Amino acid metabolism**					
Lysine	*lysA*	2	2	C	C
L-Aspartate	*ansA*	4	1	C(3)/pB(1)	C
L-Alanine	*ald*	2	2	C	C
L-Glutamate	*gltB*	1	1	C	C
	gltD	1	1	C	C
Serine	*sdaA*	2	2	C	C
Glycine	*glyA*	1	1	C	C
Threonine	*thrC*	2	3	C	C
L-Histidine	*hisD*	1	1	C	C
Proline	*proC*	3	3	C	C
Arginine	*nos*	1	1	C	C
Tyrosine	*hisC*	3	1	C(2)/pB(1)	C
Phenylalanine	*pheA*	1	1	C	C
**Organic acid metabolism**					
Citrate	*gltA*	2	3	C	C
cis-Aconitate	*acnA*	1	1	C	C

Oxaloacetate	*pyc*	1	1	C	C
	mqo	2	1	C	C
Fumarate	*fumC*	1	0	C	(-)
	fumA	1	1	C	C
	nagK	1	0	C	(-)
	fahA	1	0	pB	(-)
Acetate	*acs*	2	2	C	C
	aarC	1	0	C	(-)
	ackA	1	1	C	C
Oxalosuccinate	*icd*	1	1	C	C
L-Tartrate	*ttuC*	1	0	C	(-)
Glyoxylate	*aceA*	1	1	C	C
D-Glucarate	*glcD*	2	3	C	C
	glcE	1	1	C	C
	glcF	2	2	C	C
	gudD	1	0	C	(-)
Succinate	*sdhC*	1	1	C	C
	sdhB	1	1	C	C
	sdhA	1	1	C	C
2-Oxo-glutarate	*korA*	1	1	C	C
	korB	1	1	C	C
(S)-Malate	*mdh*	1	1	C	C
**Proton efflux**					
F-type ATPase	*atpB*	1	1	C	C
	atpC	1	1	C	C
	atpE	1	1	C	C
	atpF	1	1	C	C
	atpH	1	1	C	C
NADH dehydrogenase	*ndh*	4	3	C	C
Cytochrome c oxidase	*ctaA*	2	1	C	C
	ctaB	2	1	C	C
	coxA	5	3	C	C
	coxB	1	1	C	C
	coxC	1	1	C	C
	coxD	1	1	C	C
	qoxA	2	1	C	C
	qoxB	2	1	C	C
	qoxC	1	1	C	C
	qoxD	1	1	C	C
**Cell wall component**					
Peptidoglycan	*murA*	2	2	C	C
	murB	1	1	C	C
	murC	1	1	C	C
	murD	1	1	C	C

	murE	2	2	C	C
	murF	4	2	C(3)/pD(1)	C
	murG	1	1	C	C
	ddl	2	1	C	C
	mraY	1	1	C	C
	pbp2A	2	2	C	C
	pbpG	1	1	C	C
	mrcA	1	1	C	C
	pbpA	2	2	C	C
	pbpB	1	1	C	C
	spoVD	1	1	C	C
	vanY	4	0	C	(-)
	dgkA	1	1	C	C
	uppS	1	1	C	C
	bacA	2	3	C	C
	bcrC	8	2	C	C
Wall teichoic acid (WTA)	*tagTUV*	6	5	C	C
	wecA	1	1	C	C
Lipoteichoic acid (LTA)	*ltaS*	4	3	C	C
	ugtP	3	1	C	C
**Flagellar assembly**					
Basalbody/Hook	*fliE*	1	1	C	C
	fliF	1	1	C	C
	fliG	1	1	C	C
	fliH	1	1	C	C
	fliI	1	1	C	C
	fliK	1	1	C	C
	fliM	1	1	C	C
	fliN	1	1	C	C
	fliO	1	1	C	C
	fliR	1	1	C	C
	flgB	1	1	C	C
	flgC	1	1	C	C
	flgD	1	1	C	C
	flgE	1	1	C	C
	flgF	1	1	C	C
	flgG	1	1	C	C
	flgK	1	1	C	C
	flgL	1	1	C	C
	flhA	1	1	C	C
	flhB	1	1	C	C
Filament	*fliA*	1	1	C	C
	fliC	1	1	C	C
	fliD	1	1	C	C

	fliS	2	2	C	C
	fliT	1	1	C	C
Stator	*motA*	2	2	C	C
	motB	2	2	C	C
Regulator	*flgM*	1	1	C	C
	fliY	1	1	C	C
	rpoD	1	1	C	C
	ropN	2	0	C	(-)

Note: C means chromosome; pB means plasmid B; pD means plasmid D. (-) means no detection

Table S3. The plasmid-borne genes related to mineral dissolution of PMA2-2

Gene name	Description	Location
**Biofilm formation**		
bapA	Large surface protein	pD*
**EPS biosynthesis**		
wecB	UDP-N-acetylglucosamine 2-epimerase	pD
**Amino acid metabolism**		
ansA	Type II asparaginase	pB
hisC	Histidinol-phosphate transaminase	pB
**Organic acid metabolism**		
fahA	Fumarylacetoacetase	pB*
**Cell wall component**		
murF	YheC/YheD family protein	pD
**Two-component system**		
kinE	Sporulation sensor kinase E	pG
kinA	Sporulation sensor kinase A	pF
yesN	Response regulator	pB
liaR	Response regulator	pA
vanRC	Response regulator	pA
vanSC	HAMP domain-containing histidine kinase	pA
phoP	Alkaline phosphatase synthesis response regulator	pA
kinB	Sporulation sensor kinase B	pC
saeS	HAMP domain-containing sensor histidine kinase	pA*
devR	Response regulator	pC*
**Quorum sensing**		
nisP	Lantibiotic leader peptide-processing serine protease	pA
nisK	Lantibiotic biosynthesis sensor histidine kinase	pA
nisR	Lantibiotic biosynthesis response regulator	pA*
nisC	Lanthionine synthetase C family protein	pA*
nisT	ABC transporter ATP-binding protein	pA*
nisB	Lantibiotic dehydratase	pA*
nisA	Gallidermin/nisin family lantibiotic	pA*
comQ	Polyprenyl synthetase family protein	pA*
comP	Sensor histidine kinase	pA*

Note: * means only found in plasmid.

Table S4. Upregulated exometabolites related to amino acids in the presence of PMA2-2

Metabolite	Fold change (PMA2-2 VS PMD1-1)	Metabolite	Fold change (PMA2-2 VS PMD1-1)
Asp-Asp-Tyr	16.0***	Dityrosine	1.8***
Lys Glu Arg Phe Ala	11.3***	Phe-Gly-Gln	1.8***
Ala Ile Trp	2.9***	Cysteinyl-Valine	1.8**
Glutamylhydroxyproline	2.7***	Tyr-Glu-Thr	1.7***
Trp-Gly-Lys	2.4**	Lys-Leu-His	1.7***
Glycylprolylarginine	2.3***	Rigin	1.6***
Glu Asn Glu	2.2***	Tyrosyl-Valine	1.6***
Phe-Tyr	2.2***	Pro-Asp-Ser	1.6***
Ala-His-Lys	2.1***	N-Lactoyl-Tryptophan	1.6***
Trp-Asp-Gln	2.0***	Isoleucyllysine	1.6***
Ala-Val	2.0***	Cys-Arg-Glu-Lys-Ala	1.6***
Valylcysteine	2.0***	4-Hydroxyisoleucine	1.5***
His-His-Gln	1.9***	Epsilon-Deoxy-Fructosyl-Lysine	1.5***
Val Ala Gly Val Ala	1.5**	Phenylalanylhistidine	1.3***
Phe Ile Phe	1.5***	Ala-Thr-Trp-Leu-Pro-Pro-Arg	1.3***
N-Carboxyacetyl-D-Phenylalanine	1.5***	Asp Pro Asp	1.3***
Lys-Gly-Ser	1.5***	Val-Asp-Ile	1.3***
Ile-Trp	1.5***	Alanyl-Dl-Phenylalanine	1.3***
Gly-Tyr	1.4***	Leu-Trp	1.3**
Pyroglutamylisoleucine	1.4***	Ile-Tyr	1.3***
Leu-Tyr	1.4***	Glu-Asp-Thr	1.2***
Homocitrulline	1.4***	Tyr-Pro	1.2***
Isodityrosine	1.4***	Pro-Leu	1.2***
Phe-Val	1.4***	Val-Leu	1.2***
Tyr-His-Arg	1.4***	Val-Gly-Ile	1.2***
Glu Pro Tyr	1.4***	Kainic Acid	1.2***
Glu Ile Asp Thr Lys	1.4***	Val-Met	1.2***
Pro-Trp	1.4***	Tyrosyl-Alanine	1.2***
Pro Phe	1.4***	N-Acetylglycine	1.2***
Glu-Met	1.4***	Pro-Tyr	1.2***
Prolylhydroxyproline	1.3***	N-Lactoyl-Phenylalanine	1.2***
Glu Ala Gln	1.3***	Suberylglycine	1.2***
Ile-Val-Asn	1.3***	Ala-Glu	1.2***

Note: $^{**}p < 0.01$, $^{***}p < 0.001$

Table S5. Upregulated exometabolites related to organic acids, EPS, and cell wall components in the presence of PMA2-2

Metabolite	Fold change (PMA2-2 VS PMD1-1)	Metabolite	Fold change (PMA2-2 VS PMD1-1)
**Organic acids related metabolites**			
Glucose Lactate Acetate	2.9^{***}	Nicotinic Acid	1.3^{***}
N-Acetyl-Serylaspartic Acid	1.6^{***}	2-Aminoheptanedioic Acid	1.3^{***}
Argininosuccinic Acid	1.5^{***}	Crotonic Acid	1.2^{***}
Tubaic Acid	1.5^{***}	Isonicotinic Acid	1.2^{***}
Coumaryl Acetate	1.4^{***}	4-Ene-Valproic Acid	1.2^{***}
Indole-3-Butyric Acid	1.4^{***}	Urocanic Acid	1.2^{***}
Glucose Pyruvate	1.4^{***}	4-Aminohex-5-Ynoic Acid	1.2^{***}
Kynurenic Acid	1.3^{***}	1,4-Dihydroxy-2-Naphthoic Acid	1.2^{***}
4-Hydroxybutanoic Acid	1.3^{***}	2'-Deoxymugineic Acid	1.2^{***}
2-Aminonicotinic Acid	1.3^{***}	2-Keto-3-Deoxy-D-Gluconic Acid	1.2^{***}
3-Hydroxybutyric Acid	1.3^{***}	2-Hydroxybutyric Acid	1.1^{***}
Remifentanil Acid	1.3^{***}		
**EPS related metabolites**			
ADP Ribose	10.9^{***}	Dermatan L-Iduronate	1.6^{***}
Isorhamnetin 3-Rutinoside-7-Glucoside	3.4^{***}	DTDP-4-Acetamido-4,6-Dideoxy-D-Galactose	1.3^{***}
Fructose 1-Phosphate	2.1^{***}	3-Deoxy-D-Manno-Octulosonate	1.3^{***}
Cellulose, Microcrystalline	2.0^{***}	UDP-N-Acetyl-D-Galactosamine	1.2^{***}
Pentosan Polysulfate	1.8^{***}	Lichenin	1.2^{***}
Adenosine Diphosphate Ribose	1.7^{***}	3-O-Beta-D-Galactopyranosyl-L-Arabinose	1.2^{***}
Rutinose	1.6^{***}		
**Cell wall component related metabolites**			
Adp Ribose	10.9^{***}	Udp-N-Acetyl-D-Galactosamine	1.2^{***}
Adenosine Diphosphate Ribose	1.7^{***}	Dityrosine	1.8^{***}
Dtdp-4-Acetamido-4,6-Dideoxy-D-Galactose	1.3^{***}	Isodityrosine	1.4^{***}
3-Deoxy-D-Manno-Octulosonate	1.3^{***}	Myristic Acid	1.3^{***}

Note: $^{***}p < 0.001$

Table S6. Element concentrations, pH, and cell number in PMA2-2-inoculated medium with minerals after 12 and 24 h of incubation

Minerals		Time (h)	Dissolved Fe (μM)	Dissolved Al (μM)	Dissolved Mg (mM)	pH	Cell number (Log_{10}CFU mL^{-1})
Potassium-feldspar	No bacteria	12	(-)	0.19 ± 0.05	(-)	7.00 ± 0.01	(-)
		24	(-)	0.11 ± 0.05	(-)	6.99 ± 0.01	(-)
	PMA2-2	12	(-)	13.09 ± 1.13	(-)	4.69 ± 0.01	6.64 ± 0.10
		24	(-)	21.48 ± 2.67	(-)	4.60 ± 0.02	5.82 ± 0.07
Olivine	No bacteria	12	0.05 ± 0.02	(-)	0.89 ± 0.04	7.30 ± 0.01	(-)
		24	0.04 ± 0.00	(-)	0.89 ± 0.02	7.31 ± 0.03	(-)
	PMA2-2	12	15.24 ± 2.73	(-)	1.30 ± 0.01	4.91 ± 0.12	6.77 ± 0.10
		24	30.95 ± 0.55	(-)	1.43 ± 0.03	4.74 ± 0.03	6.17 ± 0.03
Hematite	No bacteria	12	0.04 ± 0.00	(-)	(-)	7.16 ± 0.04	(-)
		24	0.04 ± 0.00	(-)	(-)	7.12 ± 0.05	(-)
	PMA2-2	12	25.95 ± 0.90	(-)	(-)	4.69 ± 0.05	8.06 ± 0.02
		24	71.43 ± 1.56	(-)	(-)	4.66 ± 0.06	7.47 ± 0.13

Note: the values were shown as mean ± SD (n = 3), (-) means not measured.

Table S7. The compositions of minerals used in this study

Mineral	Al_2O_3	SiO_2	Fe_2O_3	CaO	MgO	K_2O	Na_2O	TiO_2
Biotite	17.10%	40.72%	13.95%	0.18%	15.88%	8.82%	0.10%	0.89%
K-feldspar	19.24%	60.57%	0.12%	0.88%	0.07%	9.87%	4.51%	0.04%
Hematite	0.48%	9.82%	86.58%	0.11%	0.06%	0.06%	0.01%	0.68%
Olivine	0.23%	41.77%	10.53%	0.08%	47.89%	0.01%	0.04%	0.01%

Supporting Figures

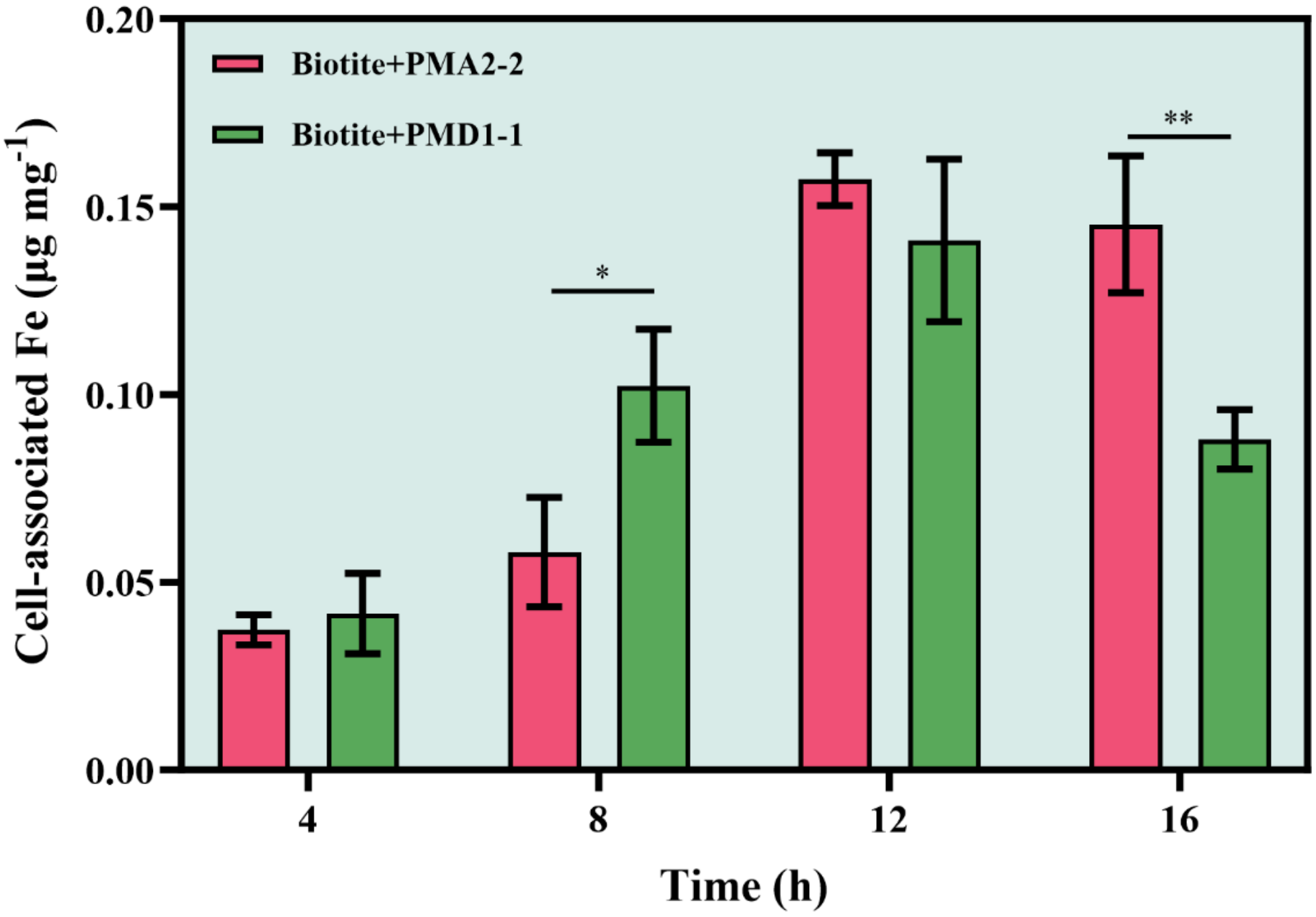

Fig. S1. Intracellular Fe in the presence of PMA2-2 and PMD1-1 for 4, 8, 12 and 16 h of incubation with biotite. Error bars represent ± one standard deviation (*n* = 3). Two-tailed *p* values for unpaired t tests are indicated between PMA2-2 and PMD1-1, *$p < 0.05$, **$p < 0.01$.

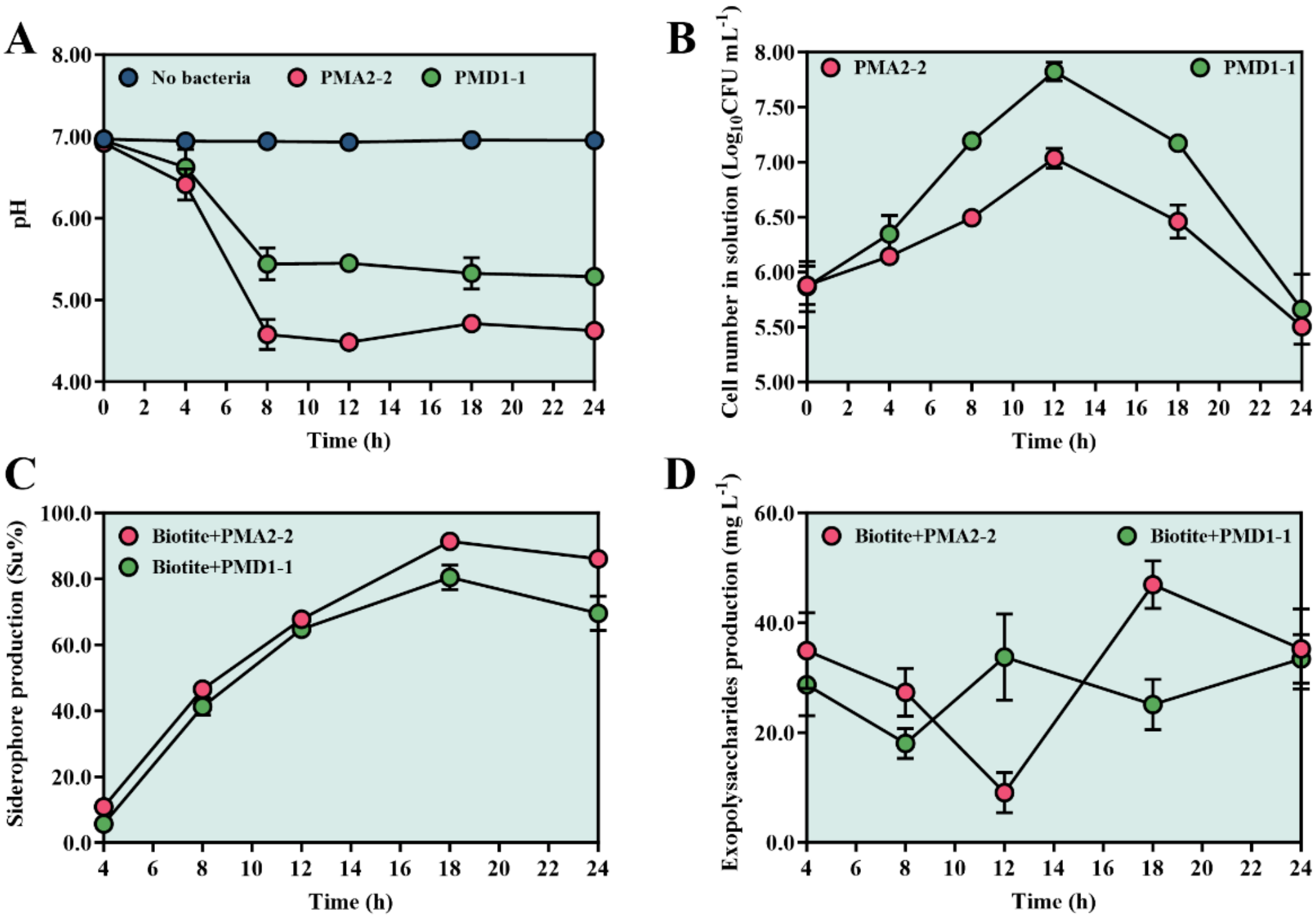

Fig. S2. pH value (A), cell number (B), siderophore (C) and exopolysaccharide (D) production in the presence of PMA2-2 and PMD1-1 for 0, 4, 8, 12, 18 and 24 h of incubation in the absence of biotite. Error bars represent ± one standard deviation (n = 3).

A

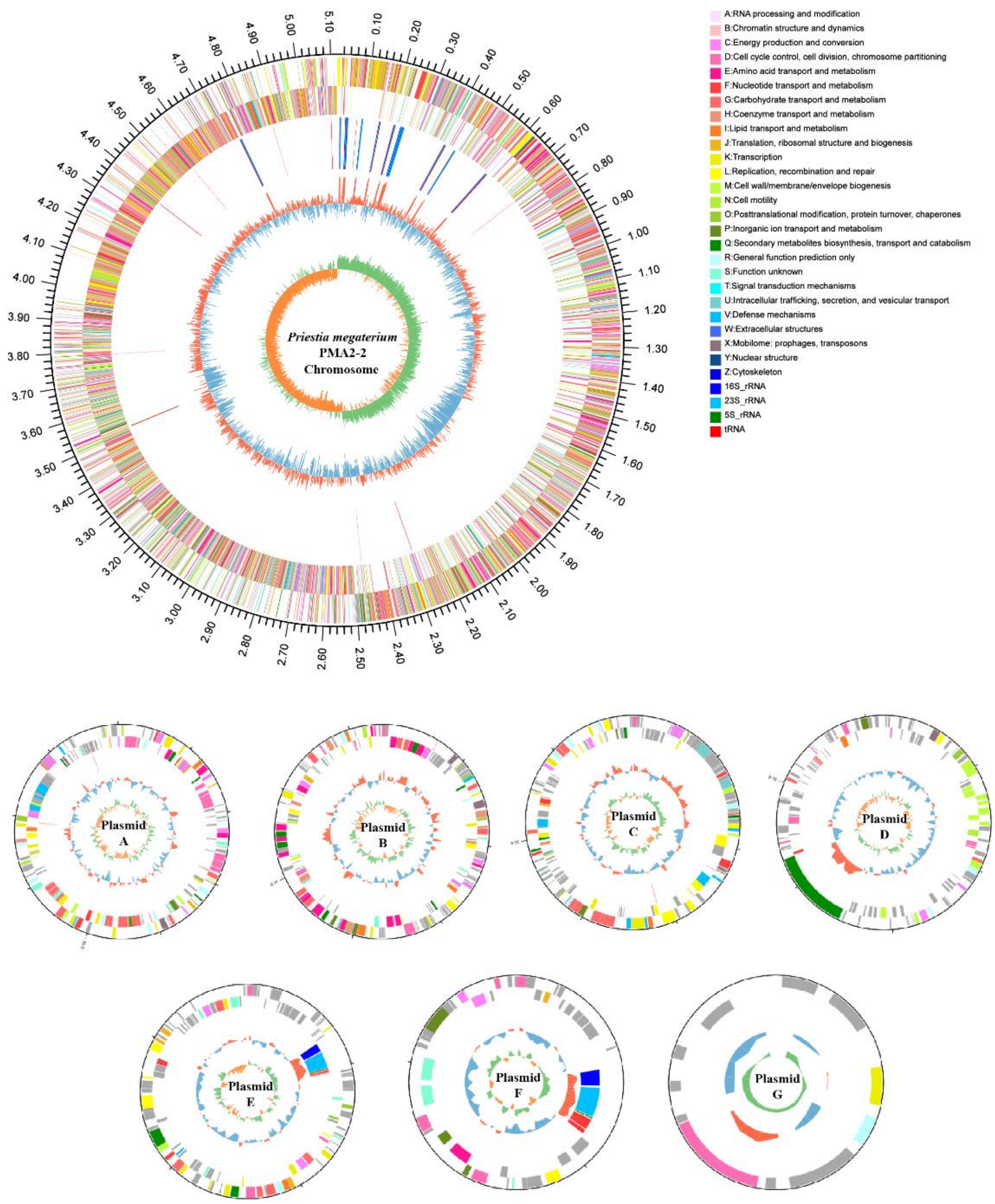

B

Assembly ID	Length (bp)	G+C (%)	Gene number
Chromosome	5119230	38.27	5181
Plasmid A	181073	34.17	181
Plasmid B	147262	34.48	169
Plasmid C	138210	33.42	151
Plasmid D	123337	33.54	107
Plasmid E	95957	34.17	102
Plasmid F	51245	36.29	43
Plasmid G	9110	34.64	11

Fig. S3. The genomic map (A) and the characteristics of chromosome and plasmids (B) of strain PMA2-2

A

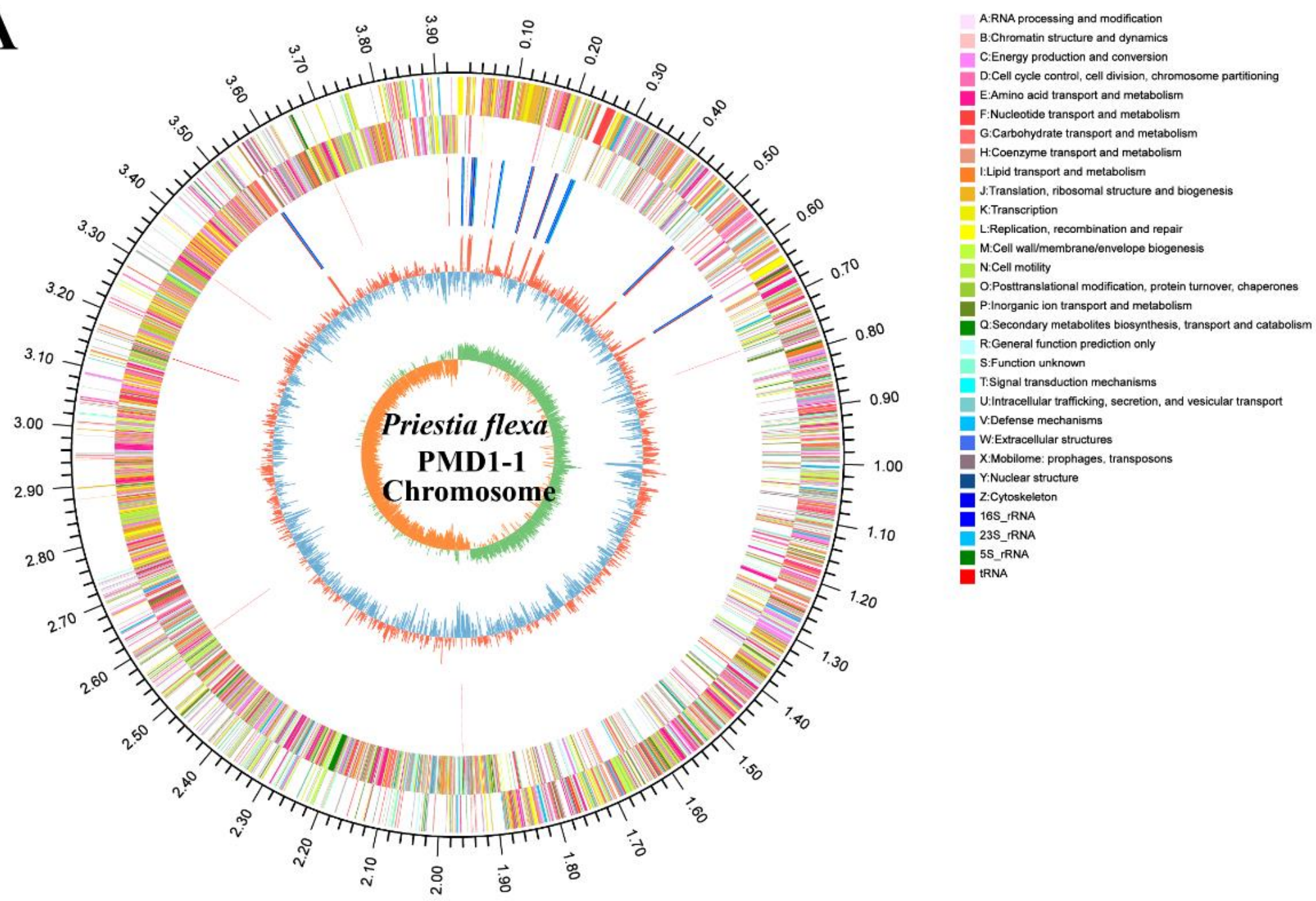

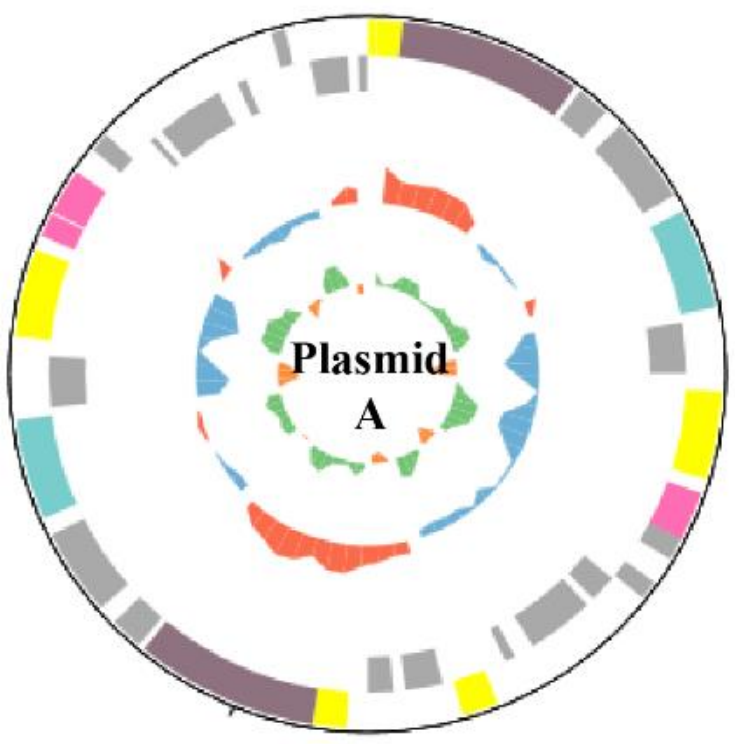

B

Assembly ID	Length (bp)	G+C (%)	Gene number
Chromosome	3936812	38.16	3981
Plasmid A	35653	32.89	35

Fig. S4. The genomic map (A) and the characteristics of chromosome and plasmids (B) of strain PMD1-1

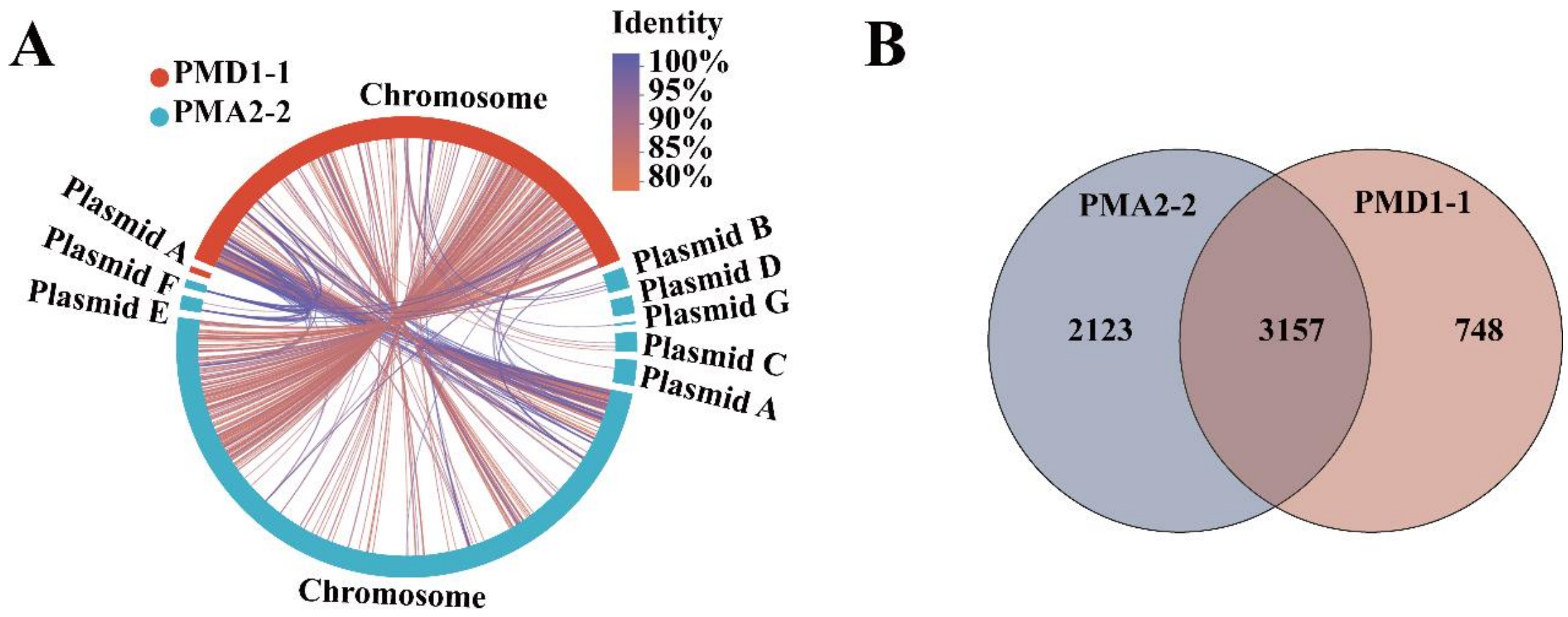

Fig. S5. Whole-genome synteny analysis of PMA2-2 and PMD1-1 (A) and homologous gene analysis of PMA2-2 and PMD1-1 (B).

A

Differentially expressed genes of two-component system		
**Signals**	**Genes**	**Log₂FC (PMA2-2 VS PMD1-1)**
Phosphate limitation	*phoRP*	3.5-4.0
Secretion stress	*cssSR*	3.9-6.5
Bacitracin	*bceSR*	4.0-4.9
Oxygen limitation	*resED*	3.2-3.7
Mg^{2+}-Citrate complex	*citST*	3.2-4.6
C4-Dicarboxyrate	*dctSR*	2.5-4.1
Malate	*malKR*	2.8-6.2
Salt stress	*degSU*	3.6-3.6
Low temperature	*desKR*	3.1-5.6
Cell wall-active antibiotics	*liaSR*	5.5-6.6
Attractant	*cheAY*	3.7-4.1
Glutamine	*glnKL*	5.1-6.2

Differentially expressed genes of quorum sensing		
**Signal molecules**	**Genes**	**Log_2FC (PMA2-2 VS PMD1-1)**
Mature antimicrobial peptide	*nisABCTPKR*	4.2-6.0
AI-2	*luxS*	3.2
ComX	*comQP*	3.1-3.7

Differentially expressed genes of second messenger		
**Signal molecules**	**Genes**	**Log_2FC (PMA2-2 VS PMD1-1)**
ppGpp	*spoT*	2.8
c-di-GMP	*sadC*	3.6
	adrA	5.1
	cheBR	3.7-4.6
c-di-AMP	*disA*	3.7
	dacA	2.5-3.4

B

Differentially expressed genes of ABC transporters		
**Substrates**	**Genes**	**Log_2FC (PMA2-2 VS PMD1-1)**
Ribose/AI-2/D-Xylose	*rbsC*	1.1
HMP/FAMP	*thiXYZ*	6.5-7.6
Spemidine/Putrescine	*potABCD*	3.6-4.7
Glycine betaine/Proline	*proVWX*	1.9-2.4
Raffinose/Stachyose/Melibiose	*msmEFGK*	2.6-5.8
Phosphate	*pstSABC*	4.4-6.8
Zinc	*znuABC*	4.6-7.1
Fe(III) dicitrate	*fecBCD*	3.1-5.7
Dipeptide	*dppBCDE*	5.5-5.9
Branched-chain amino acid	*livFGHKM*	4.6-7.7
Arginine/Lysine/Histidine	*artPQR*	3.8-4.8
Hemine	*hrtAB*	3.9-5.2

Differentially expressed genes of bacterial secretion system			
**Pathways**	**Elements**	**Genes**	**Log_2FC (PMA2-2 VS PMD1-1)**
Sec-SRP (Signal Peptide Recognition Particle)	Inner membrane protein (IMP)	*secEDFG-yajC-yidC*	2.6-3.9
	ATPase	*secA*	3.0
	SRP receptor	*ftsY*	5.1
	Targeting protein	*ffh*	3.3
Twin arginine targeting	IMP	*tatAC*	2.4-4.6

Differentially expressed genes of phosphotransferase system		
**Substrates**	**Genes**	**Log_2FC (PMA2-2 VS PMD1-1)**
Glucose	*ptsG*	1.1
Maltose	*malT*	1.6
Sucrose	*scrA*	2.3-5.2
Fructose	*fruA*	3.1

C

Differentially expressed genes of carboxylation		
**Cofactor**	**Genes**	**Log_2FC (PMA2-2 VS PMD1-1)**
Biotin	*bioABCDFH*	4.1-5.5
	fabFGIZ	2.8-6.4

D

Differentially expressed genes of cell wall component		
**Components**	**Genes**	**Log_2FC (PMA2-2 VS PMD1-1)**
Peptidoglycan	*murABCDEFG*	1.2-6.0
	ddl	3.0
	mraY	3.4
	pbp2A-pbpG-mrcA	3.3-4.0
	pbpAB-spoVD	3.6-4.2
	vanY	3.1-4.2
	dgkA	3.6
	uppS	3.5
	bacA-bcrC	3.0-5.0
Wall teichoic acid (WTA)	*wecA*	4.0
	tagATUV	2.9-5.8
Lipoteichoic acid (LTA)	*ugtP*	3.5-5.7
	ltaS	4.2-4.8

Fig. S6. Significantly upregulated genes in PMA2-2 compared with those in PMD1-1 involved in environmental response regulation (A), transport (B), carboxylation (C), and cell wall component (D).

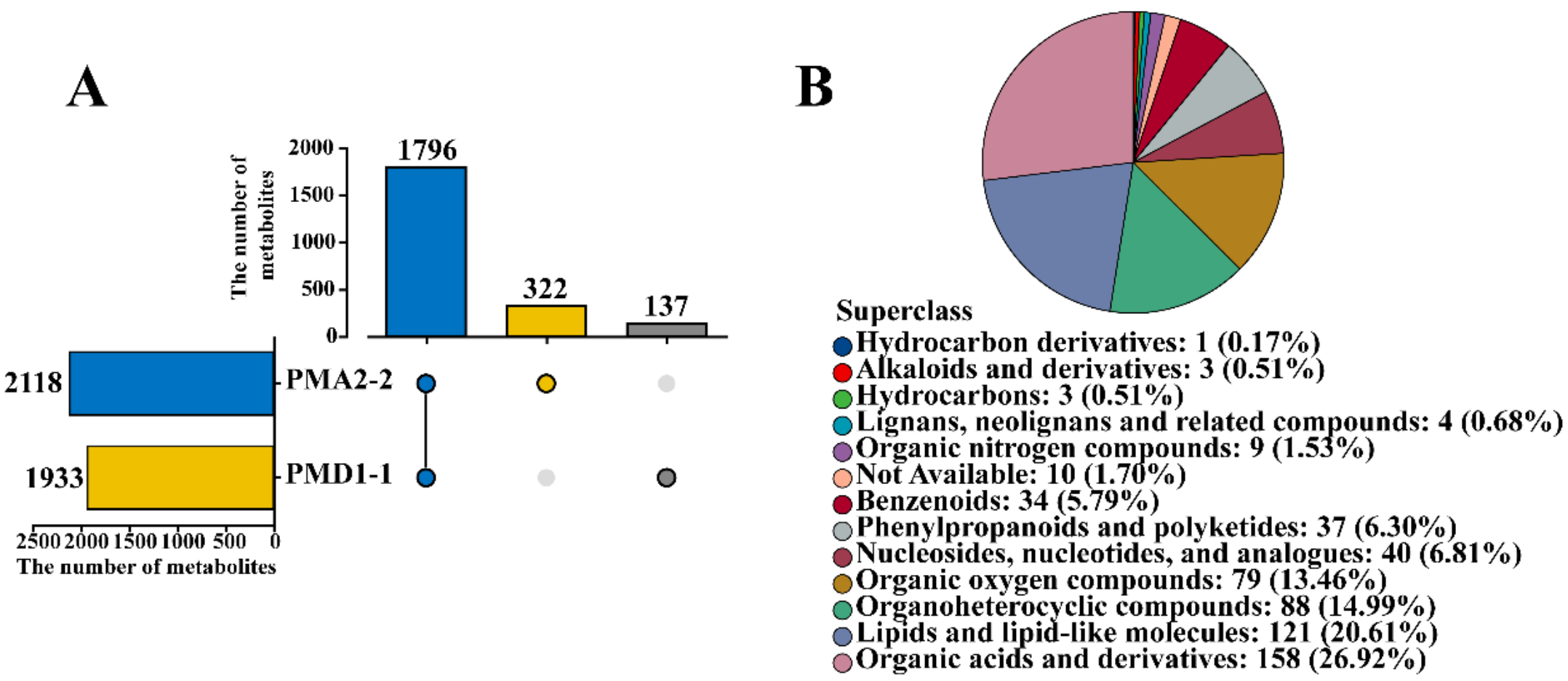

Fig. S7. Common and distinctive metabolites between PMA2-2 and PMD1-1 (A), the blue dot represents the exometabolites that both found in the presence of PMA2-2 and PMD1-1, the yellow dot represents the exometabolites only found in the presence of PMA2-2, while the gray dot represents the exometabolites only found in the presence of PMD1-1. HMDB compound classification diagram of differential metabolites (B).

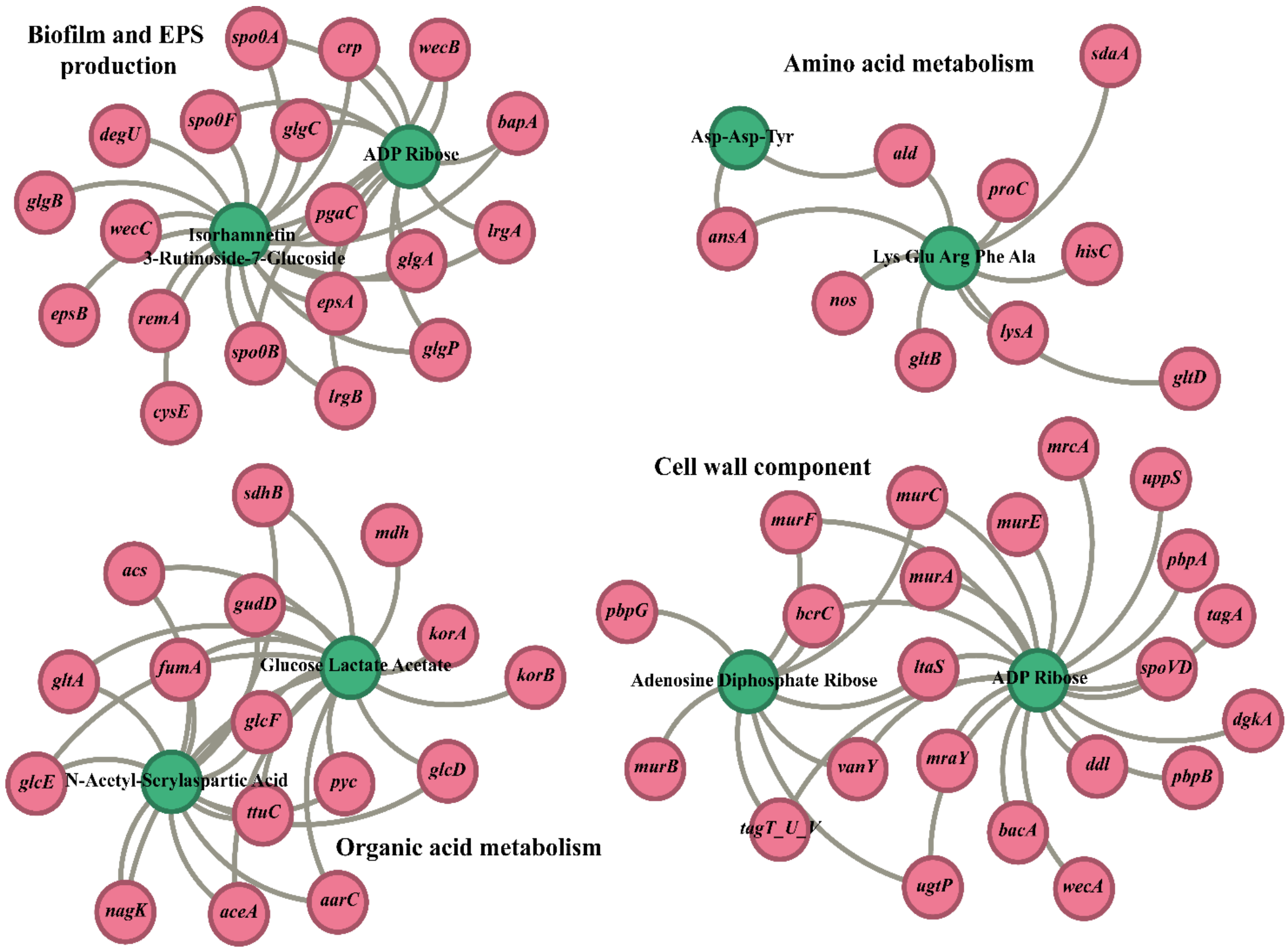

Fig. S8. Multi-omics analysis of interaction network between mineral-dissolution related genes and metabolites. Green spheres represent metabolites that are positively correlated with genes (depicted as red spheres). Statistical analysis was conducted using Spearman's correlation to evaluate the relationship between metabolite data and gene expression data. The correlation values were all greater than 0.8 with p values < 0.05.

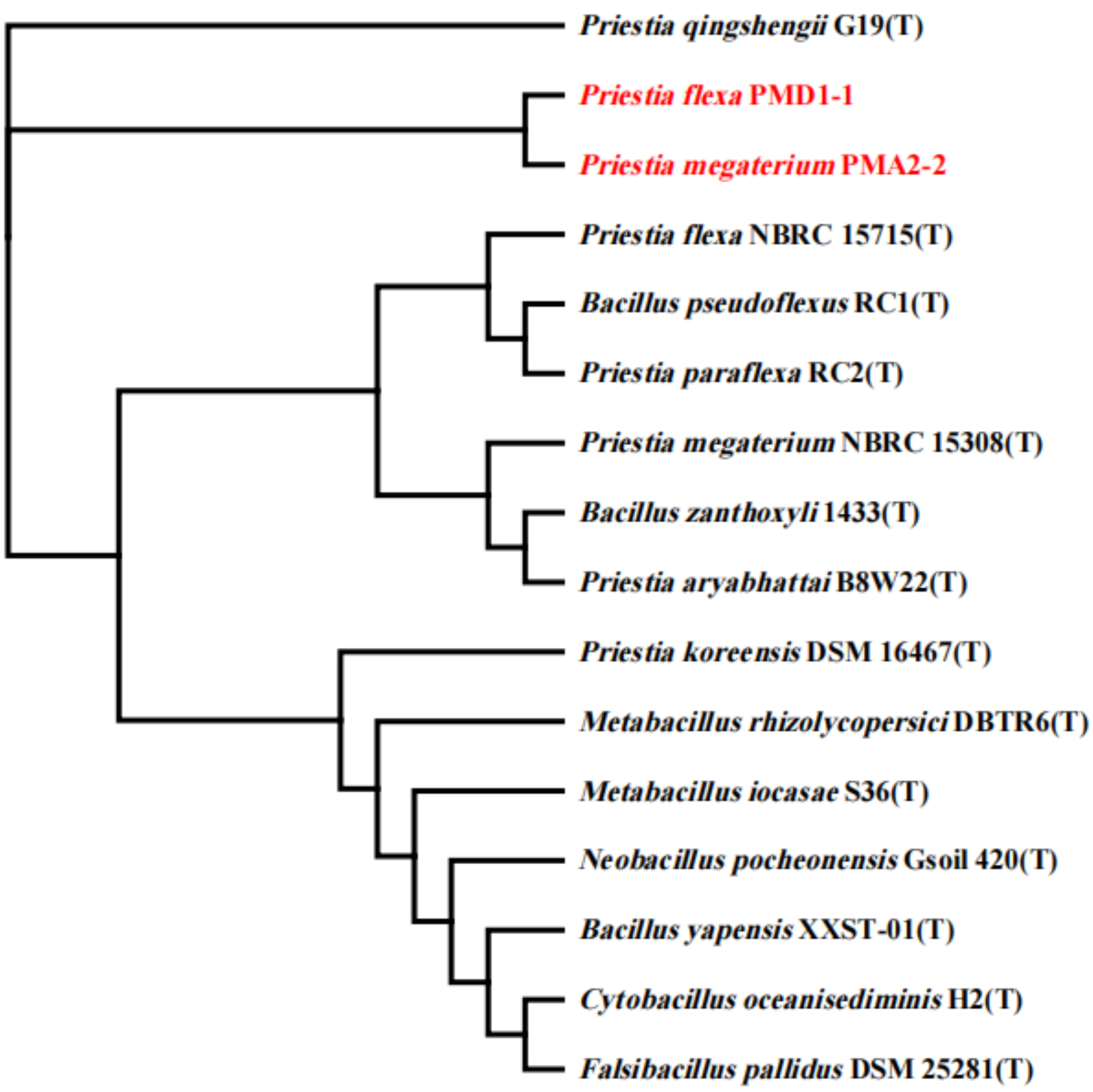

Fig. S9. The phylogenetic tree of strain PMA2-2 and PMD1-1
